# A Collision Coupling Model Governs the Activation of Neuronal GIRK1/2 Channels by Muscarinic-2 Receptors

**DOI:** 10.3389/fphar.2020.01216

**Published:** 2020-08-12

**Authors:** Shai Berlin, Etay Artzy, Reem Handklo-Jamal, Uri Kahanovitch, Hanna Parnas, Nathan Dascal, Daniel Yakubovich

**Affiliations:** ^1^Department of Neuroscience, Rappaport Faculty of Medicine, Technion, Haifa, Israel; ^2^Department of Physiology and Pharmacology, Sackler Faculty of Medicine, Tel-Aviv University, Tel Aviv-Yafo, Israel; ^3^Silberman Institute of Life Sciences, Hebrew University, Jerusalem, Israel; ^4^Department of Neonatology, Schneider Children’s Hospital, Petah Tikva, Israel

**Keywords:** collision-coupling, G-protein cycle, kinetic model, GIRK channel, G-Protein Coupled Receptor

## Abstract

The G protein-activated Inwardly Rectifying K^+^-channel (GIRK) modulates heart rate and neuronal excitability. Following G-Protein Coupled Receptor (GPCR)-mediated activation of heterotrimeric G proteins (Gαβγ), opening of the channel is obtained by direct binding of Gβγ subunits. Interestingly, GIRKs are solely activated by Gβγ subunits released from Gα_i/o_-coupled GPCRs, despite the fact that all receptor types, for instance Gα_q_-coupled, are also able to provide Gβγ subunits. It is proposed that this specificity and fast kinetics of activation stem from pre-coupling (or pre-assembly) of proteins within this signaling cascade. However, many studies, including our own, point towards a diffusion-limited mechanism, namely collision coupling. Here, we set out to address this long-standing question by combining electrophysiology, imaging, and mathematical modeling. Muscarinic-2 receptors (M2R) and neuronal GIRK1/2 channels were coexpressed in *Xenopus laevis* oocytes, where we monitored protein surface expression, current amplitude, and activation kinetics. Densities of expressed M2R were assessed using a fluorescently labeled GIRK channel as a molecular ruler. We then incorporated our results, along with available kinetic data reported for the G-protein cycle and for GIRK1/2 activation, to generate a comprehensive mathematical model for the M2R-G-protein-GIRK1/2 signaling cascade. We find that, without assuming any irreversible interactions, our collision coupling kinetic model faithfully reproduces the rate of channel activation, the changes in agonist-evoked currents and the acceleration of channel activation by increased receptor densities.

## Introduction

GIRK (G protein-activated Inwardly Rectifying K^+^-channel) channels play fundamental physiological roles, such as control of heart rate and neuronal excitability, and are also highly implicated in disease such as addiction, depression, bipolar disorder, and cardiac arrhythmias (Dascal, 1997; Hibino et al., 2010; Luscher and Slesinger, 2010; Voigt et al., 2014; Mayfield et al., 2015; Rifkin et al., 2017). Opening of the channel is achieved by a highly recognized signaling cascade involving agonist binding to a G-protein Coupled Receptor (GPCR), which in turn activates heterotrimeric G-proteins (of the Gα_i/o_-type), to promote ‘release’ of Gβγ subunits. Then, direct interaction of Gβγ subunits with GIRK leads to channel opening and the appearance of the agonist-evoked current, I_evoked_ [(Logothetis et al., 1987; Reuveny et al., 1994; Lim et al., 1995; Slesinger et al., 1995); reviewed in (Clapham and Neer, 1997; Dascal and Kahanovitch, 2015)].

Despite more than four decades of studies, the details behind this prototypical activation scheme remain highly debated. For instance, it remains unclear how the different signaling proteins are arranged at the membrane to bring about robust and efficient channel opening, fast activation kinetics and, importantly, signaling specificity (the strong preference for GIRK activation by Gβγ derived from G_i/o_ rather than G_s_ or G_q_). Two contrasting mechanisms have been proposed [reviewed in (Hein and Bunemann, 2009)]. The first, denoted collision coupling (Tolkovsky and Levitzki, 1981; Tolkovsky et al., 1982; Shea and Linderman, 1997; Shea et al., 1997; Shea et al., 2000), assumes unrestricted diffusion of GPCRs, G-proteins, and effectors in the plasma membrane. After GPCR activation, GIRK activation occurs through random collisions with proteins of this cascade (Vorobiov et al., 2000; Hein et al., 2005; Touhara and MacKinnon, 2018). According to this model, a single receptor may activate several G-proteins (as in visual system; (Arshavsky et al., 2002)) and, therefore, an increase in the number of receptors is expected to accelerate activation kinetics of I_evoked_. Indeed, this was observed for several GPCR-GIRK cascades (Vorobiov et al., 2000; Wellner-Kienitz et al., 2000; Hein et al., 2005; Kahanovitch et al., 2017). The second mechanism posits long-lived “preformed” complexes of GPCRs, G-proteins, regulatory proteins (e.g., Regulators of G Protein Signaling; RGS) and GIRKs in various combinations (Huang et al., 1995; Lavine et al., 2002; Fowler et al., 2006; Jaen and Doupnik, 2006; Dupre et al., 2007; Rusinova et al., 2007; Dupre et al., 2009; Nagi and Pineyro, 2014; Tateyama and Kubo, 2018). In support, several lines of evidence indicate that Gβγ and Gα_i/o_ can associate with GIRKs, as early as in the endoplasmic reticulum ((Rebois et al., 2006; Robitaille et al., 2009); reviewed in (Zylbergold et al., 2010)), recruit G-proteins to the plasma membrane (Rishal et al., 2005; Kahanovitch et al., 2014), and possibly remain associated at the plasma membrane (Fowler et al., 2006; Rebois et al., 2006; Riven et al., 2006; Berlin et al., 2011; Kano et al., 2019). Further, in neurons, cardiomyocytes and heterologous expression models, some GIRKs exhibit an agonist-independent (basal) current (I_basal_) that is Gβγ-dependent, suggesting some kind of pre-coupling of GIRK with the G protein before the receptor has been engaged (reviewed in (Dascal and Kahanovitch, 2015)). We have also shown that GIRK1/2, but not the GIRK2 homotetramers, recruit G-proteins to the plasma membrane, favoring Gβγ over Gα (Rishal et al., 2005; Kahanovitch et al., 2014). The preferential association with Gβγ explains the high, Gβγ-dependent I_basal_ of GIRK1/2, contrasting the small and Gβγ-independent I_basal_ of GIRK2 homotetramers (Rubinstein et al., 2009; Yakubovich et al., 2015). Together, these findings support the existence of dynamic G protein-GIRK complexes; however, whether they require permanent association is debated (Yakubovich et al., 2015). It is conceivable that different signaling cascades may proceed at different modes, namely collision or preformed modes, or a mixture of the two (e.g., only G protein and effector are pre-coupled).

The preformed complex model can seamlessly account for specificity (i.e., preferential activation of the channel by Gβγ released from a particular type of Gα-subunit) as well as for the high speed of GPCR-induced activation of GIRKs (Hille, 1992; Huang et al., 1995), limited mainly by the kinetics of G protein cycle (Vorobiov et al., 2000; Lohse et al., 2008; Hein and Bunemann, 2009). However, specificity can also be quantitatively described in purely kinetic terms, i.e. collision coupling (Touhara and MacKinnon, 2018). For instance, if a particular Gα-type, namely Gα_i/o_, is quicker to provide Gβγ to the channel than other Gα’s, it would appear as though the channel solely responds to Gβγ derived from that specific Gα (Touhara and MacKinnon, 2018). Indeed, heterologous overexpression of proteins of the β-adrenergic-Gα_s_ cascade can lead to activation of GIRK *via* Gα_s_-derived Gβγ (Lim et al., 1995; Bender et al., 1998; Touhara and MacKinnon, 2018). These results show that the system can indeed proceed, at least in some instances, *via* a collision coupling mechanism. Lastly, at high levels of expression of the reactants (proteins participating in the cascade), especially the GPCR, the kinetics and magnitude of effector activation *via* a collision coupling cascade would be indistinguishable from those attained by a preformed complex (Lauffenburger and Linderman, 1996). Therefore, the nature and concentrations of the reactants strongly affect the speed and specificity of the responses, thereby making it hard to distinguish between different modes of activation. It is therefore critical to study these mechanisms by systematic “titration” of the interactors.

In the current work, we set out to understand the mode of coupling in the classical M2R-Gα_i/o_-GIRK cascade, by combining electrophysiological, fluorescence, and biochemical measurements in *Xenopus* oocytes with kinetic modeling. Specifically, we studied this cascade by systematically varying and quantifying surface densities of proteins involved in it, and monitored outcomes on GIRK activation. Next, we combine a Thomsen-Neubig-like mathematical model of GPCR activation (Thomsen and Neubig, 1989; Zhong et al., 2003) with extant models of GIRK activation by Gβγ to quantitatively describe GIRK activation in detail. We find that a collision coupling model faithfully reproduces both the fast activation kinetics of agonist-induced GIRK responses, and their dependence on GPCR surface density.

## Methods

### Ethical Approval and Animals

Experiments were approved by Tel Aviv University Institutional Animal Care and Use Committee (permits M-08-081 and M-13-002). All experiments were performed in accordance with relevant guidelines and regulations. *Xenopus laevis* female frogs were maintained and operated as described (Dascal and Lotan, 1992). Briefly, frogs were kept in dechlorinated water tanks at 20 ± 2°C on 10 h light/14 h dark cycle, anesthetized in a 0.17% solution of procaine methanesulphonate (MS222), and portions of ovary were removed through a small incision in the abdomen. The incision was sutured, and the animal was held in a separate tank until it had fully recovered from the anesthesia and then returned to post-operational animals’ tank. The animals did not show any signs of post-operational distress and were allowed to recover for at least 3 months until the next surgery. Following the final collection of oocytes, after 4 surgeries at most, anesthetized frogs were killed by decapitation and double pithing.

### DNA Constructs and mRNA Injection

cDNA constructs of GIRK subunits, Gβ_1_, Gγ_2_, M2R, YFP-GIRK1 were described in detail in previous publications (see (Tabak et al., 2019) for a detailed list). Fluorescent proteins (CFP and YFP) contained the A207K mutation to prevent dimerization (Zacharias et al., 2002). DNAs of M2R-CFP and M2R-YFP were produced by inserting the PCR product of the human M2R (Lechleiter et al., 1990), flanked by EcoRI on both sides, in pGEM-HJ vector containing CFP (cerulean) or YFP flanked by EcoRI and HindIII, yielding M2R-C/YFP_CT_. The M2R-Gα_i3-C351G_ tandem cDNA was created by ligating the M2R cDNA sequence in frame with the Gα_i3-C351G_ cDNA, connected *via* a 6 nucleotide sequence GAATTC (EcoRI restriction site). Thus, the full primary sequences of M2R and Gα_i3-C351G_ are connected by a 2-amino acid linker, Glu-Phe. The DNA of GluR1_L507Y_ (Stern-Bach et al., 1998) was generously provided by Y. Stern-Bach (Hebrew University). All DNAs were cloned into pGEM-HE, pGEM-HJ or pBS-MXT vectors, which are high expression oocyte vectors containing 5’ and 3’ untranslated sequences of *Xenopus* β-globin (Liman et al., 1992), as previously described (Rishal et al., 2005; Berlin et al., 2011). mRNA was transcribed *in vitro* as described in (Dascal and Lotan, 1992) and precipitated overnight at -20°C with 4 M LiCl. mRNAs were divided into 1 to 2 μl aliquots and stored at -80°C. 50 nl of mRNA were injected into the equatorial part of oocytes, two to three days before the experiments.

### Electrophysiology

Whole-cell GIRK currents were measured using two-electrode voltage clamp (TEVC) with Geneclamp 500 (Molecular Devices, Sunnyvale, CA, USA), sampled at 1 kHz and filtered at 200 Hz, at room temperature (21-23°C), as previously described (Rubinstein et al., 2009), using agarose cushion electrodes (Schreibmayer et al., 1994) filled with 3M KCl, with resistances of 0.1–0.6 MΩ. Data acquisition and analysis were done using pCLAMP software (Molecular Devices). For recording, oocytes were placed in a fast-perfusion chamber (see **Figure S1A** and below). Holding potential was set to -80 mV. Basal GIRK currents (I_basal_) were measured by switching from physiological solution (low K^+^, ND96: 96 mM NaCl, 2 mM KCl, 1 mM CaCl_2_, 1 mM MgCl_2_, 5 mM Hepes, pH 7.5) to a high K^+^ solution (high K^+^, in mM: 24 KCl, 2 NaCl, 1 CaCl_2_, 1 MgCl_2_, HEPES, pH 7.5). For recording evoked currents (I_evoked_), solution was then switched to high K^+^ solution containing 10 μM acetylcholine (ACh). Addition of 5 mM Ba^2+^ (blocker of GIRK) was typically applied at the end of each recording to isolate non-GIRK currents and to calculate net GIRK currents. Total current (I_total_) was assessed by summing basal and evoked currents (I_total_= I_basal_+ I_evoked_).

In the perfusion system employed in these experiments, each perfusion tube (inlet) is directly incorporated into the bath chamber (shaped like a thin elongated bar), rather than *via* a manifold, ending ~3 mm from the oocyte in large diameter openings (see **Figure S1A**). The suction has also been incorporated directly into the bath, 1 mm above the level of the oocyte, to reduce the bath solution volume and to allow fast exchange of the solution. In order to access the solution exchange time, we employed an AMPA receptor (AMPAR) mutant; GluR1_L507Y_, which lacks fast desensitization (Stern-Bach et al., 1998). The activation time constant (τ_act_) of AMPAR activation is below 1 ms (Grosskreutz et al., 2003) and can therefore be considered essentially instantaneous compared to the slower kinetics of GIRK activation. 1 ng RNA of GluR1_L507Y_ was injected into *Xenopus* oocytes. 50 nl of 20 mM solution of EGTA was injected 2 hours before experiment, to prevent the appearance of Ca^2+^-dependent Cl^-^ currents (Dascal, 1987). AMPAR was activated by applying saturating glutamate concentration (1 mM) to the bathing solution. A representative recording of AMPAR current is shown in **Figure S1B**. The rising phase of the response to glutamate, fitted to a single exponential function, was 88.6 ± 14.5 ms (n=9), indicating that the average solution exchange rate time constant of our perfusion system was about 90 ms.

### Whole Oocyte Radioactive Quinuclidinyl Benzilate (QNB*) Labeling

Whole oocyte binding experiments were performed as described in (Ben-Chaim et al., 2003). Briefly, three days following mRNAs injection, oocytes were dropped into 200 μl of 0.67 nM QNB* (Halvorsen and Nathanson, 1981). After 1 min of incubation, the oocyte was taken to a washing chamber that contained 4000 μl of ligand-free solution (washing stage) and rapidly (after 1–2 s) removed to the scintillation liquid by use of a custom device. The device is composed of plastic holder that enables insertion of a pipetor with a standard pipette tip (volumes up to 200 μl) trimmed 4 mm from the edge. This ensures extraction of the oocyte with minimal amount of liquid. Then, individual oocytes were placed in vials to which 4 ml of scintillation fluid was added and counted with Packard 2100TR TriCarb Liquid Scintillation Analyzer. Specific binding was determined by subtracting the binding from native, uninjected oocytes.

### Fluorescence Imaging

Imaging of fluorescence in the plasma membrane (PM) of whole oocytes was performed as previously described (Berlin et al., 2010; Berlin et al., 2011). Briefly, whole oocytes were placed in a glass-bottom dish, and all images were obtained from the animal hemisphere close to oocyte’s equator (see Berlin et al, 2010; **Figure 2**-micrographs showing homogenous fluorescence in the animal pole). Imaging experiments were performed on a confocal laser scanning microscope (LSM 510 Meta, Zeiss, Germany) with 20x or 5x objective lenses, digital zoom = 2, pinhole 3 Airy units, equipped with a HFT 405/514/633 beam splitter. CFP was excited by 405 nm and emission was collected in the wavelength interval of 449-500 nm, peak emission 481 nm; YFP was excited by 514 nm and emission collected in the interval of 524-609 nm, peak emission: 534 nm. Analysis was done using Zeiss LSM software. The net intensity of fluorescence in the PM was measured by averaging the signal obtained from three standard regions of interest along the membrane with background subtraction (Berlin et al., 2010). In each experiment, uninjected oocytes were tested for intrinsic fluorescence with the use of either lasers: 405 and 514 nm excitation. In all confocal imaging procedures, care was taken to completely avoid saturation of the signal. In each experiment, all oocytes from the different groups were studied using constant LSM settings.

### Statistical Analysis

Statistical analysis was performed using SigmaPlot software (Systat Software, San Jose, California, USA). Data are presented as mean ± S.E.M. Two group comparisons were done using two-tailed student’s t-test. Multiple group comparison was done using one-way analysis of variance (ANOVA), with *post hoc* Tukey tests. Activation kinetics (τ_act_) were obtained by fitting evoked currents by a mono-exponential fit.

### Modeling and Simulation

Steady state calculations for estimation of initial conditions were done with Matlab for Windows (Mathworks Inc., Natick, Massachusetts). System of algebraic equations is shown in **Supplemental Material 2** and was solved numerically. Time-course simulation was done utilizing Berkeley Madonna 8.3.23.0 (R. Macey and G. Oster, University of California, Berkeley) for Windows. System of ordinary differential equations was generated based on schemes shown in **Figures 4A, B** and **Figure 5B**, and solved numerically by the 4^th^ order Runge-Kutta method.

### Estimation of GIRK-Gβγ Interaction Parameters

Reported GIRK-Gβγ affinity values span several orders of magnitude depending on estimation method (nM – mM range, (Dascal, 1997)). Considering this we utilized available crystal structure of GIRK2-Gβγ (Protein Data Bank number 4KFM, (Whorton and MacKinnon, 2013)) and two structures of GIRK1- Gβγ complex generated by homology modeling and protein-protein docking procedure (Mahajan et al., 2013) for structure-based prediction of protein-protein interaction free energy and affinity. In particular, the above mentioned structures were submitted to PRODIGY server (Xue et al., 2016) that calculated both free energy of interaction and K_D_ values utilizing algorithm which is based on correlation between number of interfacial contacts at the interface of a protein–protein complex and its experimental binding affinity together with properties of the non-interacting surface as described by Kastritis et al. (2011). The k_on_ (association rate constant values) were predicted utilizing Transcomp software (Qin et al., 2011). This software utilizes transient-complex theory developed by Alsallaq and Zhou (2008) for predicting protein-protein association rate constants. Coordinates supplied by structural data supplied in PDB format are used to generate the transient complex and rate constant is calculated based on the electrostatic interaction energy in the transient complex.

## Results

### Collision Coupling Between M2R and G_i/o_ in GIRK Cascade in *Xenopus* Oocytes

**Figure 1:**
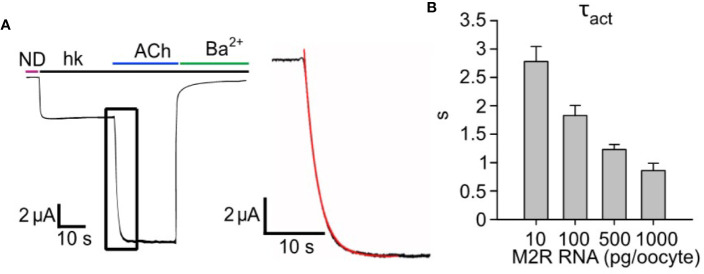
Increasing expression levels of M2R accelerates the activation of GIRK1/2. **(A)** – a representative GIRK1/2 activation experiment. Oocytes were injected with the following RNAs: GIRK1 and GIRK2 (2 ng/oocyte each) and 500 pg/oocyte M2R. I_evoked_ was elicited by 10 µM ACh. Inset- zoom-in on the activation phase of I_evoked_ (black plot) and a mono-exponential fit (red). **(B)** – kinetics of GIRK1/2 activation. Oocytes expressed a constant amount of GIRK1/2 (2 ng RNA/oocyte), with increasing levels of M2R, in the range 10-1000 pg/oocyte, and τ_act_ was determined by monoexponential fitting as shown in A (N=2-7 experiments, n= 13-25 cells).

In previous publications, we presented evidence for catalytic collision coupling between M2R and Gα_z_ (Vorobiov et al., 2000) and GABA_B_ receptors with endogenous or coexpressed Gα_i/o_ (Kahanovitch et al., 2017) in the GPCR-G protein-GIRK cascade reconstituted in *Xenopus* oocytes. To examine whether this is also the case for the M2R-G_i/o_-GIRK cascade, and for the following quantitative description and kinetic modeling of the cascade, we first characterized the mode of M2R-G_i/o_ coupling using our previously developed strategy (Vorobiov et al., 2000). Specifically, we initially assessed how changes in GPCR (M2R) concentration impacts the kinetics of activation (τ_act_) of I_evoked_ of heterotetrameric GIRK1/2 channels. Here, we used a saturating concentration of acetylcholine (ACh; 10 µM) to activate different densities of ectopic M2R, whereas the signal transduction from GPCR to the channel relied on endogenous G_i/o_ proteins. We employed a fast perfusion system with a time constant of solution exchange below 100 ms (see *Methods* and **Figure S1**). Importantly, in neurons and cardiomyocytes the τ_act_ of I_evoked_ is in the range 200-700 ms (Pott, 1979; Sodickson and Bean, 1996). Thus, our measurements of τ_act_ in the oocyte introduces an overestimation of GIRK1/2 activation kinetics. However, this overestimation is relatively minor, especially at low densities of M2R (see below). Our results show that the increase in the amount of mRNA of M2R per oocyte (i.e., increase in surface density) speeds up the activation of GIRK1/2 (**Figure 1A**, red plot- mono-exponential fit from which we extract τ_act_), with a corresponding decrease in the time constant of activation (**Figure 1B**). These results are consistent with those obtained for M2R-G_z_-GIRK and GABA_B_R-G_i/o_-GIRK cascades in this heterologous model (Vorobiov et al., 2000; Kahanovitch et al., 2017).

### Estimation of Membrane Protein Density

For detailed kinetic analysis of GIRK1/2 activation, we sought to estimate the densities of proteins involved in our system, explicitly GIRK1/2, M2R and G-protein subunits- Gα and Gβγ, by a quantitative approach previously developed in our lab (Yakubovich et al., 2015). Briefly, channel density is typically calculated based on the maximal GIRK1/2 current (i.e., I_βγ_) measured in oocytes that coexpress Gβγ at saturating concentration (usually 5 ng/oocyte of Gβ RNA and 1-2 ng Gγ RNA). Under these conditions, the channel’s open probability (P_o_) is ~ 0.105. Channel density in M2R-expressing oocytes can also be calculated from the total current obtained upon activation with saturating 10 µM dose of ACh (I_total_). I_total_ is the sum of agonist-independent GIRK1/2 current, I_basal_, and the ACh-elicited I_evoked_. We found that, for GIRK1/2, there is a stable relationship between I_total_ and I_βγ_ over a wide range of channel densities such that, on average, I_βγ_ = 2I_total_ (Yakubovich et al., 2015). If I_βγ_ or I_total_ are known, GIRK1/2 density could be estimated using a modification of the classical equation (Hille, 2002):

$$\text{Eq}.1\;{\text{I}}_{\beta \gamma }=2\cdot {\text{I}}_{\text{total}}={\text{i}}_{\text{single}}\cdot {\text{P}}_{\text{o}}\cdot \text{N}$$

where i_single_ is the single channel current and N is the number of functional channels in the PM. The channel’s surface density is defined as N/S, where S is the surface area of the cell (210^7^ µm^2^, deduced from oocyte’s capacitance of ~200 nF (Dascal, 1987)). Based on data and calculations from (Yakubovich et al., 2015), under the conditions used here (24 mM K^+^ external solution), the surface density for GIRK1/2 or YFP-GIRK1/GIRK2 can be estimated using a simple translation factor:

$$\text{Eq}.2\;\text{density}(\text{channels}/\mu {\text{m}}^{2})=0.79\;{\text{I}}_{\beta \gamma }\;(\mu \text{A})\;=1.58\;{\text{I}}_{\text{total}}(\mu \text{A})\;$$

**Figure 2:**
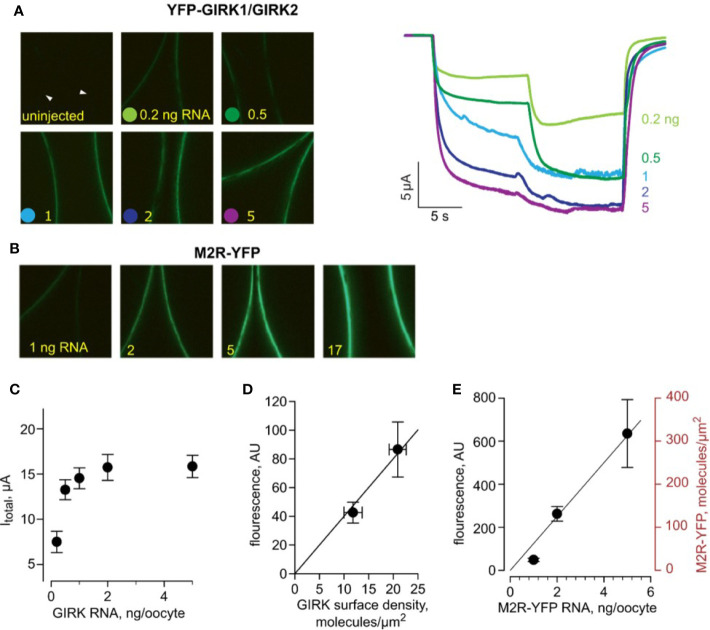
Estimating the surface density of M2R-YFP. All data are from one experiment. **(A)** - (left) Representative micrographs of oocytes (membrane at equator) expressing equal amounts of RNAs of YFP-GIRK1 and GIRK2, and 1 ng M2R (wt). Injected YFP-GIRK1 mRNA amounts are indicated. (right) Representative currents from oocytes from the same experiment (from the same groups as in left panels). Note the gradual increase in I_basal_ and I_total_, reaching a plateau at 2 ng/oocyte of channel’s RNA. The current measurements in this experiment were done with a slower perfusion rate and were not included in the kinetic analysis. **(B)** – Representative micrographs of oocytes, injected with the indicated amounts of M2R-YFP, imaged at the same day and under identical settings as those in A. **(C)** – I_total_ of YFP-GIRK1/GIRK2 channels as a function of channel’s RNA dose. **(D)** – calibration of the surface density of YFP. YFP-GIRK1 fluorescence (in arbitrary units, AU) is plotted versus channel density induced by the two lowest doses of GIRK RNA (<1 ng/oocyte), within the linear range of fluorescence-current relationship. Channel density was calculated from I_total_ as explained in the text. The correlation between fluorescence and number of YFP-GIRK1 molecules is shown with superimposed linear regression line, extended to origin of coordinates. The regression equation was y=4x, i.e. one channel/µm^2^ corresponds to fluorescence intensity of 4 AU. Note that, since each channel has two YFP molecules, the calibration factor in this experiment is: 1 YFP molecule/µm^2^ = 2 AU. **(E)** –estimating the surface density of M2R-YFP, for RNA concentrations of 1, 2 and 5 ng/oocyte. YFP fluorescence, in AU, is shown on the left Y-axis. M2R-YFP surface density (right axis) was calculated using the calibration factor derived from YFP-GIRK1/GIRK2 measurements. The relationship between M2R-YFP RNA dose and M2R-YFP surface density was fitted with linear regression, extended to the origin of coordinates, in the form y=62.5x.

In most experiments reported here, oocytes were injected with 1-2 ng or GIRK1 and GIRK2 m-RNA each. This generally elicited strong channel activity that corresponds to a “high density” group with an average surface density of ~21 GIRK1/2 channels/µm^2^ (Yakubovich et al., 2015). There is a possibility of formation of functional GIRK2 homotetramers under these experimental conditions. However, the basal current we measured ranged between 3 to 5 µA (see **Figure 2A**). In oocytes, injection of 1 ng mRNA of GIRK2 gives rise to basal currents of 0.05 - 0.2 µA (Rubinstein et al., 2009). Therefore, under our experimental conditions, it is most likely that the predominant channels recorded are indeed GIRK1/2 heterotetramers. Moreover, the preferred stoichiometry of the homologous GIRK1/4 channel is two subunits of GIRK1 and two subunits of GIRK4, rather than GIRK4 homotetramers (Silverman et al., 1996).

Next, we employed YFP-GIRK1/GIRK2 as a “molecular ruler” to translate surface densities of the channel, obtained from current, to fluorescence measurements. Here, YFP density was assumed to be twice that of the channel, since each GIRK1/2 heterotetramer is believed to contain two GIRK1 subunits, by analogy with GIRK1/4 (Silverman et al., 1996; Corey et al., 1998). First, we determined the conditions for optimal channel expression for the calibration procedure. We injected increasing amounts of YFP-GIRK1, GIRK2 and Gβγ RNAs and observed a linear relationship between I_βγ_ and YFP-GIRK1 fluorescence over the range of channel RNA doses of 0.2-1 ng/oocyte, in line with the assumption that fluorescence corresponds to functional channels (**Figure S2**). Linearity was lost at high RNA doses, suggesting that at high expression levels, some channels are non-functional (possibly not at membrane). In the experiment shown in **Figure 2**, we expressed a range of doses of YFP-GIRK1/2 with 1 ng M2R RNA and measured surface levels of channel fluorescence and total GIRK currents in response to ACh (**Figures 2A, C, D**). We also expressed a range of doses of M2R-YFP and monitored YFP surface levels (**Figures 2B, E**). A linear relationship between YFP-GIRK surface density and I_total_ of YFP-GIRK1/GIRK was observed at low doses of RNA (below 1 ng) and this range was used for the estimation of YFP-GIRK1 density for calibration purposes (**Figure 2D**). In the same experiment and with identical imaging settings, we measured the PM expression of M2R-YFP at different RNA doses and, using the calibration factor from **Figure 2D**, calculated the PM density of M2R-YFP. **Figure 2E** shows that the relationship between the RNA dose and M2R-YFP density was linear in the range 1 – 5 ng RNA, yielding receptor densities of ~20 to ~300 M2R molecules/µm^2^.

There are reports showing that the density of ion channels can be higher at the animal hemisphere or enriched around the injection site, which may effectively increase the density of PM proteins (M2R) in these areas (Robinson, 1979; Lopatin et al., 1998; Machaca and Hartzell, 1998). The assumption of homogeneity of M2R and GIRK distribution is therefore an approximation, which gives very good agreement with experiment. Note that, even if all the receptors and channels are located exclusively in the animal hemisphere, the surface density will only be changed two-fold.

### Quantifying the Relationship Between M2R-YFP Surface Density and GIRK1/2 Activation Parameters

**Figure 3:**
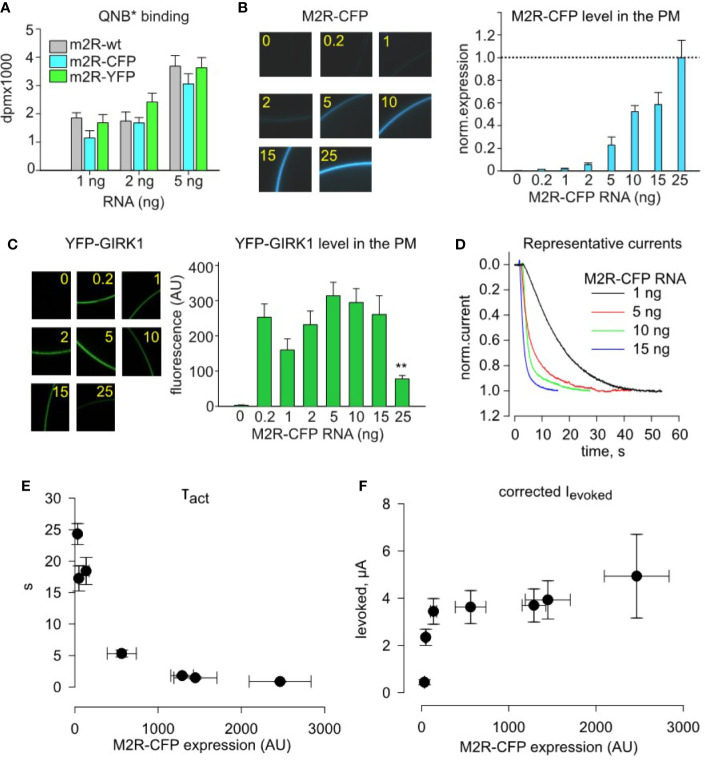
Quantitative analysis of dose-dependency of τ_act_ and amplitude of I_evoked_ on plasma membrane density of M2R-CFP. **(A)**-Whole cell radioligand-labeling by quinuclidinyl benzilate (QNB*) shows that identical doses of injected RNA produce similar surface expression of M2R, M2R-CFP and M2R-YFP, for three different amounts of receptor RNAs. B-F, quantitative analysis of incremental expression of M2R-CFP reveals collision-coupling activation of YFP-GIRK1/GIRK2 channels. Oocytes were injected with constant mRNA amounts of YFP-GIRK1 and GIRK2 (1 ng each) but with increasing doses of M2R-CFP RNA. **(B)** – RNA dose-dependent increase in the surface levels of M2R-CFP. Representative micrographs of oocytes (left) expressing m2R-CFP and summary of expression (right). RNA amounts of M2R-CFP RNA, in ng/oocyte, are indicated in yellow. n=7-12 oocytes in each group. **(C)** – expression of M2R-CFP does not affect the surface level of YFP-GIRK1/GIRK2 except for the decrease at the highest dose of M2R-CFP, 25 ng/oocyte (**, p<0.01). Left panel shows representative micrographs of YFP-GIRK1/GIRK2 – expressing oocytes. Numbers within the images indicate the amounts of M2R-CFP RNA, in ng/oocyte. Right panel shows summary of expression data (n=7-12 oocytes in each group). **(D)** – representative normalized I_evoked_, elicited by 10 µM ACh in oocytes injected with the indicated amounts of M2R-CFP RNA. For simplicity, only the initial (activation) phase of I_evoked_ is shown. **(E, F)** – τ_act_ is reduced **(E)** and I_evoked_ amplitude **(F)** is increased with increased surface density of M2R-CFP. AU, arbitrary units. τ_act_ and I_evoked_ data are from cells exemplified in B – D; n=5-12 in each group.

To compare the PM expression of different M2R constructs used in this study, we injected a range of RNA doses of wild-type (*wt*) M2R, M2R-YFP and M2R-CFP and measured the number of QNB binding sites in the PM of intact oocytes utilizing the methodology developed by Ben-Chaim et al. (Ben-Chaim et al., 2003). All three M2R constructs rendered similar number of QNB binding sites (**Figure 3A**), showing that they express at similar levels. We could therefore extend the M2R-YFP RNA – density relationship shown in **Figure 2E** towards M2R*wt* and M2R-CFP.

We next studied the impact of M2R-CFP surface density on activation parameters of YFP-GIRK1/GIRK2. We expressed a range of M2R-CFP receptor densities (with a constant amount of channel’s RNA), by injecting 1-15 ng RNA/oocyte, and monitored the PM level of M2R-CFP (**Figure 3B**) and YFP-GIRK1 (**Figure 3C**) along with currents amplitude and τ_act_ of I_evoked_ (**Figures 3D–F**). The level of YFP-GIRK1/GIRK2 remained unchanged at all doses of M2R RNA except when the receptor was injected at excessively high doses, 25 ng. This yielded a decrease in the PM level of the channel (**Figure 3C**), likely due to non-specific competition of RNAs for the same pool of ribosomes (Richter and Smith, 1981) or a trafficking interference. We have, therefore, adjusted the amplitude of I_evoked_ for the change (even if slight) in channel’s PM level (**Figure 3F**). Together, we find that increase in M2R density (validated by fluorescence) is associated with a sharp rise in both the speed and amplitude of channel activation (**Figures 3D–F**). Notably, maximal amplitude of I_evoked_ is obtained at lower PM densities of the receptor than those required to obtain the fastest activation (lowest τ_act_; compare **Figures 3E**, **F**). These observations are in-line with the predictions of the catalytic collision coupling model. Unexpectedly, we note that, though M2R-CFP expresses at equivalent levels as M2R*wt* (**Figure 3A**), it exhibits slower kinetics at all RNA doses (compare **Figures 1D**, **3D–F**). Therefore, M2R-CFP was solely used to assess surface density of the receptor, but not for quantitative description of the native cascade, where we use M2R*wt*.

### Modeling: G Protein Cycle Model

**Figure 4:**
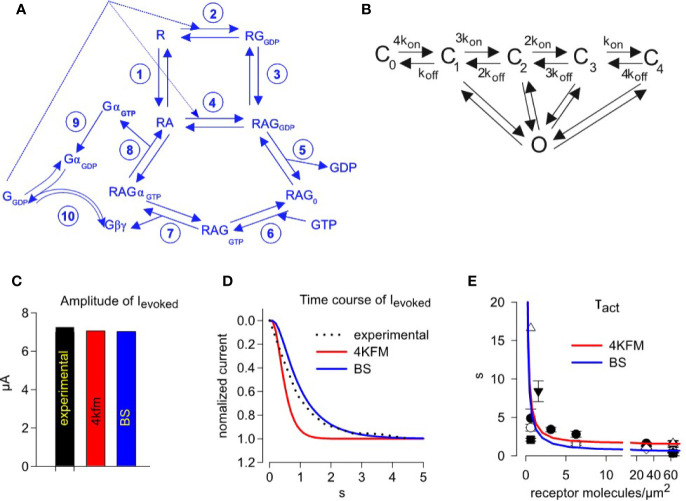
Simulating activation kinetics and its dependence on M2R surface density. **(A)** – scheme of the G-protein cycle. **(B)** – graded contribution model of GIRK1/2 activation. Rate constants of reactions shown in A and B are summarized in **Table 1**. **(C)** –comparison of the experimentally observed and predicted ACh-evoked currents, with GIRK-Gβγ interaction parameters from two structural models, 4KFM and BS. **(D)** - representative analysis of the time course of GIRK1/2 activation. The experimental result (dotted black line) is from an oocyte injected with 0.5 ng M2R RNA. Superimposed are simulated currents according to graded contribution model of GIRK1/2 activation. The experimental parameters in this cell were: I_basal_ = 15.5 µA, I_total_= 22.8 µA. Estimated channel density was 36 channels/µm^2^. Initial concentrations of Gα and Gβγ available to the channel are: for the case of 4KFM model 3.23 molecules Gα_i_/channel and 0.45 molecules Gβγ/channel; for the case of BS model, 3.24 molecules Gα_i_/channel and 0.46 molecules Gβγ/channel. Each plot represents the recorded or simulated current normalized to its maximum. **(E)** – mean τ_act_ values from all experiments with wild-type M2R, superimposed on data obtained from fitting of simulated time-courses according to different models. Each experimental point shows mean value of τ_act_ ± SEM from one experiment (n=3-7 oocytes). Simulated time courses were generated for the case described by Yakubovich et al. as “high density group”, i.e. I_basal_ =13.36 µA, I_total_ =17.2 µA, n=21 channels/µm^2^. Amounts of available Gα and Gβγ molecules per one channel, that are required to obtain the observed I_evoked_, were calculated using the graded contribution model: with the BS structure-based parameters, 3.65 Gβγ and 0.39 Gα molecules/channel; with the for 4kfm structure-based parameters, 3.62 Gβγ and 0.38 Gα molecules/channel.

For quantifying the M2R-G_i/o_-GIRK1/2 cascade, we elaborated the Thomsen et al. (1989) model of G-protein cycle by combining it with the ternary complex model developed by De Lean et al. (1980). Of note, a similar approach was used by Falkenburger et al. for the description of another muscarinic receptor and cascade, namely the M1R-G_q_-phosphoinositide signaling mechanism and regulation of the KCNQ channels (Falkenburger et al., 2010; Hille et al., 2014). A schematic representation of the G-protein cycle model is shown in **Figure 4A**. List of reactions with corresponding rate constants is shown in **Table 1**. To maintain microscopic reversibility, we incorporated into the G-protein cycle model a G-protein dissociation step (i.e. RGα_GTP_Gβγ dissociation, reaction 7) and also a GPCR-independent dissociation step (reaction 10) (Sarvazyan et al., 1998; Yakubovich et al., 2005). It must be emphasized that the rate constants for the latter reaction have been incorporated in the model of Touhara and MacKinnon (2018). Gα_GTP_Gβγ dissociation rate has been reported by Hollins et al. (2009) and association rate could be estimated based on the microscopic reversibility assumption (**Table 1**, reaction 7). Furthermore, GDP/GTP exchange is split into two reversible reactions. Rate constants of GDP and GTP binding have been determined experimentally (Higashijima et al., 1987; Zhong et al., 2003). We also incorporated GαGβγ nucleotide free state in the process which leads from Gα_GDP_ bound state to GTP bound state as proposed by Ross (Ross, 2008).

**Table 1:** Rate constants of GPCR mediated GIRK activation.

Reaction	k_on_ M^-1^s^-1^	k_off_ s^-1^	Reference	Calculation
1. R + A ⟺ RA	3.33∙10^2^ (k_1f_)	7∙10^-3^ (k_1b_)	(Schreiber et al., 1985)	
2. R+G_GDP_⟺RG_GDP_	5.51∙10^6^ (k_2f_)	1.6∙10^-1^ (k_2b_)		(*)
3. RG_GDP_ + A ⟺ RAG_GDP_	4.5∙10^6^ (k_3f_)	4.87∙10^-1^ (k_3b_)	(Schreiber et al., 1985)	
4. RA+G_GDP_⟺ RAG_GDP_	3.68∙10^8^ (k_4f_)	5. = 5∙10^-2^ (k_4b_)	(Ilyaskina et al., 2018)	(**)
5. RAG_0_+GDP⟺ RAG_GDP_	10^6^ (k_5b_)	5 (k_5f_)	(Zhong et al., 2003)	
6. RAG_0_+GTP⟺ RAG_GTP_	10^6^ (k_6f_)	.1 (k_6b_)	(Zhong et al., 2003)	
7. RAGα_GTP_ +Gβγ⟺ RAG_GTP_	10^6^ (k_7b_)	15 (k_7f_)	(Hollins et al., 2009)	(**)
8. RA+Gα_GTP_⟺ RAGα_GTP_	10^7^ (k_8b_)	2 (k_8f_)	(Zhong et al., 2003)	
9. Gα_GTP_→ Gα_GDP_		.02 (k_9f_)	(Zhong et al., 2003)	
10. Gα_GDP_ + Gβγ ⟺ G_GDP_	0.7∙10^6^ (k_10f_)	0.0013 (k_10b_)	(Sarvazyan et al., 1998)	
C_n_+ Gβγ⟺CGβγ_n+1_	(4-n)∙0.23∙10^6^	(n+1)∙0.037	Best scoring model, (Mahajan et al., 2013)	(*)
	(4-n)∙1.01∙10^6^	(n+1)∙0.14	4KFM, (Whorton and MacKinnon, 2013)	

Notations in brackets are used in ordinary differential equations shown in **Supplemental Material 2**.

(*) k_on_ and k_off_ estimated by PRODIGY and TRANSCOMP.

(**) k_on_ adjusted, see text.

In order to estimate kinetic parameters of reactions of the model that have not been determined experimentally, we used the open-source software tools PRODIGY for the estimation of K_D_ values (Xue et al., 2016) and TRANSCOMP for estimating association rate constants (Qin et al., 2011). Lastly, we scrutinized the crystal structure of M2R with heterotrimeric G-protein (PDB: 6OIK) (Maeda et al., 2019) for deriving the kinetic parameters related to M2R-G protein coupling (**Table 1**).

### Modeling: Channel Activation Model

We made use of our previously described GIRK1/2 gating model (Yakubovich et al., 2015), denoted “graded contribution model” (**Figure 4B**). This model is based on the assumption that a channel that is occupied by 1 to 4 Gβγ molecules can reach the open conformation, but the contribution of each Gβγ-occupied state is different; the higher Gβγ occupancy, the higher the contribution to open probability. This model is based on studies of Sadja et al. and Ivanova-Nikolova et al. (Ivanova-Nikolova et al., 1998; Sadja et al., 2002) on a highly homologous channel; GIRK1/4. To estimate the parameters of interaction between GIRK1/2 and Gβγ for the graded contribution model, we proceeded in a similar approach as done for the unknown parameters of M2R-G-protein interaction, namely we analyzed two structural models of GIRK-Gβγ complex. The first is the crystal structure of GIRK2 in complex with Gβγ (Whorton and MacKinnon, 2013) (PDB: 4KFM) and the second is a docking model for the GIRK1-Gβγ complex (Mahajan et al., 2013) (termed “best scoring model”, or BS). Both models were subjected to analysis by PRODIGY and TRANSCOMP software from which we obtained K_D_ and k_on_ values of GIRK-Gβγ interaction. For calculating initial values for all channel and G-proteins states (i.e. before agonist application), a system of algebraic equations was numerically solved assuming that the reaction between GIRK-G protein is in steady-state (**Supplemental Material 1**). Time-course of activation of GIRK1/2 was simulated as a solution of system of ordinary differential equations (**Supplemental Material**, **Eq. 19–34**).

### Simulation of GIRK1/2 Activation Time-Course and Amplitude

In order to validate our model, we simulated the experimentally observed GIRK1/2 activation by a step application of 10 µM ACh. We chose a representative recording in which 0.5 ng/oocyte M2R mRNA was injected. This corresponds to a PM expression of about 31 receptors/µm^2^, according to our calibrations (see **Figure 2E**). We used available values of Gα and Gβγ for the activation of GIRK from its I_basal_ and I_total_ as previously described (Yakubovich et al., 2015) (see **Supplemental Material 1**). We then simulated the evoked current of GIRK1/2 and compared with experimental values (**Figures 4C, D**). We find that our developed model satisfactorily reproduces the fast kinetics and the amplitude of I_evoked_, with kinetic parameters of GIRK-Gβγ interaction obtained from both structural models tested (4KFM and BS).

Subsequently, we ran time-course simulations over a wide range of M2R receptor densities, 0.1 – 100 receptors/µm^2^. The initial parameters of I_basal_ and I_total_ for these simulations were adopted from the “high density group” of GIRK1/2 channel expression obtained by injection of 1-2 ng (21.7 channels/µm^2^, I_basal_ = 13.36 µA and I_total_ = 17.2 µA; (Yakubovich et al., 2015)). In each simulation, τ_act_ was extracted from mono-exponential fit of the activation phase of I_evoked_, and the calculated values of τ_act_ versus receptor density were superimposed on the experimentally measured τ_act_ obtained from a large number of experiments with M2R*wt* (**Figure 4E**). We find that that our model satisfactorily predicts the acceleration of activation rate with the increase in GPCR density. It also faithfully reproduces the real kinetics of the receptor, namely it reproduces the fastest kinetics of activation obtained when using high receptor densities.

### Modeling Kinetics of M2R-Gα_i3_ Fusion Protein

**Figure 5:**
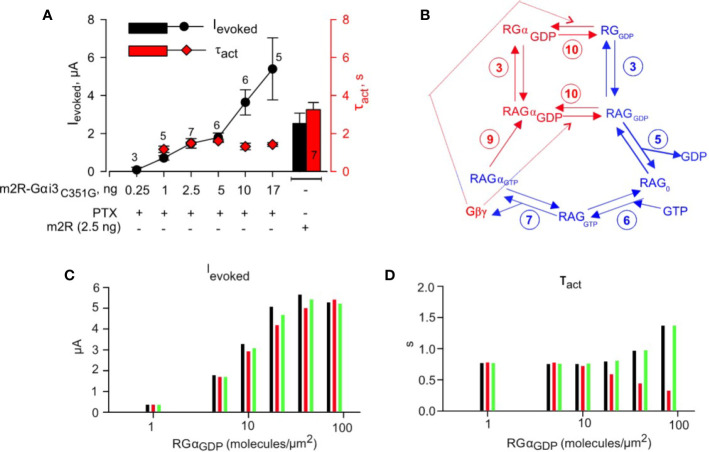
Physical tethering of M2R and Gαi3 converts a collision coupling mechanism to a preformed-complex: experiment and simulation. **(A)** – Incremental expression of fused M2R-Gα_i3_C351G (PTX-insensitive) in the presence of coexpressed A-protomer of pertussis toxin (PTX; 0.2 ng RNA/oocyte) shows increase in I_evoked_ with growing amounts of injected RNA (black plot), but kinetics of activation remain unchanged (red plot). Right- Histogram of evoked currents (black) and τ_act_ (red) of GIRK1/2 coexpressed with M2R*-wt*. Result shown are from one experiment; number of cells (n) tested in each group are shown above experimental points or in the bar. No significant change in τ_act_ was found (one way ANOVA, P = 0.154). Spearman correlation coefficient calculated for analysis of evoked currents ~ 1, P = 0.0167. **(B)** – scheme of G-protein utilized to simulate GIRK1/2 activation by M2R-Gα_i3_C351G. Blue arrows and numbering denote reactions that are shared with M2R wt activation pathway, as described in **Figure 4**. Red arrows denote reactions present only in the current scheme. The numbering of reactions and the rate constants are the same as in **Table 1**. **(C)** – simulated I_eoked_ values obtained assuming a range of expression level of fused M2R-Gα_i3_C351G. **(D)** – summary of τ_act_ obtained from fitting time-course of activation of GIRK1/2 by range of M2R-Gα_i3_C351G densities with mono-exponential function. Three possible scenarios were simulated for analysis of M2R-Gα_i3C351G_ experiments. Black bars; M2R-Gα concatemer is assumed to have same affinity to Gβγ as Gα, and no change in Gβγ concentration is assumed with concatemer expression. Red bars; M2R-Gα concatemer is assumed to have same affinity to Gβγ as Gα, and 1:1 increase in Gβγ concentration is assumed with concatemer expression. Green bars; M2R-Gα concatemer is assumed to have 10-fold lower affinity to Gβγ than Gα and no change in Gβγ concentration is assumed with concatemer expression. For simulation it was assumed that GIRK1/2 is expressed at levels similar to “intermediate density group” described in Yakubovich et al. (2015) i.e. under pre-expression conditions there are ~ 9.7 channels/µm^2^ and 3.5 Gβγ molecules/channel. It is also assumed that under PTX expression conditions most endogenous Gα_i3_ is ADP-ribosylated and subsequently degraded.

We were curious as to how would our model behave, and how would the simulated results look, should we be able to force some players to be in complex with each other. We have previously achieved forcing the precoupling of GPCR to Gα by fusing M2R to Gα_z_ (tandem) (Vorobiov et al., 2000). Notably, we found that the kinetics of GIRK activation were independent of the concentration of the tandem protein (see *Introduction*). Here, we similarly made a tandem protein consisting of M2R and Gα_i3_. Our initial tests showed that this tandem was functional and, furthermore, engendered faster activation than that obtained by the M2R-Gα_z_ tandem (**Figure 5A**). This was expected because of the much slower G protein cycle kinetics of Gα_z_ (Vorobiov et al., 2000). We also introduced the C351G mutation into Gα_i3_ to impart pertussis toxin resistance (West et al., 1985). This allowed us to silence endogenous Gα_i/o_ by expressing the A protomer of pertussis toxin, to avoid incidental collision coupling of the tethered M2R to non-tethered endogenous Gα_i/o_βγ and the ensuing Gβγ activation of GIRK (Vorobiov et al., 2000). We find that the activation kinetics of I_evoked_ were remarkably stable and independent on the doses of M2R-Gα_i3-G351G_ (**Figure 5A**, red plot), whereas the amplitude of I_evoked_ persistently increased with higher RNA dose of M2R-Gα_i3-G351G_ (**Figure 5A**, black plot). These results confirm that increase in RNA dose of the tandem is accompanied by an increase in its surface density and, more importantly, indicate that low doses of the tethered receptor-Gα pair cannot provide enough Gβγ to activate the large number of channels. We then proceeded to develop a scheme of GPCR-Gα_i3_ tandem-mediated activation of GIRK1/2 (**Figure 5B**) and subsequently simulated time-course of GIRK1/2 activation by a range of GPCR-Ga_i3_ tandem densities. Three possible scenarios were simulated: 1) M2R-Gα concatemer was assumed to have the same affinity to Gβγ as Gα, and no change in Gβγ concentration was assumed with concatemer expression; 2) M2R-Gα concatemer was assumed to have same affinity to Gβγ as Gα, and 1:1 increase in Gβγ concentration was assumed with concatemer expression; 3) M2R-Gα concatemer was assumed to have 10 fold lower affinity to Gβγ than Gα and no change in Gβγ concentration was assumed with concatemer expression. Simulated evoked currents and τ_act_ values show that, whereas I_evoked_ values increase with the increase in GPCR-Gα_i3_ density, τ_act_ values remain constant (**Figures 5C, D**). These simulations thereby fully recapitulate the outcomes on channel activation *via* a preformed complex between the GPCR and the G protein and provide a unique and contrasting picture than what we obtain when all components are free (i.e., non-fused). Thus, these observations further support the collision-coupling nature of M2R to G protein signaling in the M2R-GIRK cascade reconstituted in *Xenopus* oocytes.

### Comparison to Cooperative Gating Model

We next tested another detailed model of the GPCR-GIRK cascade based on collision-coupling published by Touhara and MacKinnon (2018), termed here cooperative gating model. This model incorporates the Thomsen et al. model of the G-protein cycle (Thomsen et al., 1988; Thomsen and Neubig, 1989), and the receptor independent G-protein heterotrimer dissociation (Sarvazyan et al., 1998; Sarvazyan et al., 2002). This model assumes that channel activation is cooperative, i.e., each Gβγ binds to the channel with stronger affinity than the previous one. It also assumes that GIRK can open only when all four Gβγ-binding sites have been occupied. In the model, we applied affinities and rate constants from (Touhara and MacKinnon, 2018), including K_D_ of binding of the first Gβγ to GIRK of 60 µM, with a cooperativity factor µ=0.3 for the binding of each next Gβγ, to generate a system of differential equations analogous to Eq. 19-34 for the simulation of GIRK1/2 activation. The densities of Gβγ and Gα were as for standard conditions, specifically ~21 channels/µm^2^ and 31 M2R receptors/µm^2^ (as for simulation of “high density group” (Yakubovich et al., 2015); 0.5 ng/oocyte RNA of M2R corresponds to ~ 31 receptors/µm^2^; see **Figure 2**).

Since GIRK1/2 has a substantial GPCR-independent but Gβγ-dependent I_basal_ [see (Rubinstein et al., 2009) for details], a certain excess of free Gβγ over Gα in the vicinity of the channel must be assumed (Yakubovich et al., 2015), whatever the mathematical approach used to describe channel’s behavior. Using the cooperative gating model equations (Eq.14-18) and parameters of GIRK-Gβγ interaction, as described by (Touhara and MacKinnon, 2018), we calculate that ~7 free Gβγ molecules are needed to account for the I_basal_ measured at “high density”. We next tested various pairs of Gβγ and Gα densities and selected those which most closely recapitulated experimental measures for I_basal_ and I_total_ (**Figure S3**). Furthermore, for analysis, we selected the minimal number of Gβγ (out of tested pairs), with the corresponding amount of Gα molecules per channel that best reproduced the experimental data (**Figure S3**, **see legend**).

**Figure 6:**
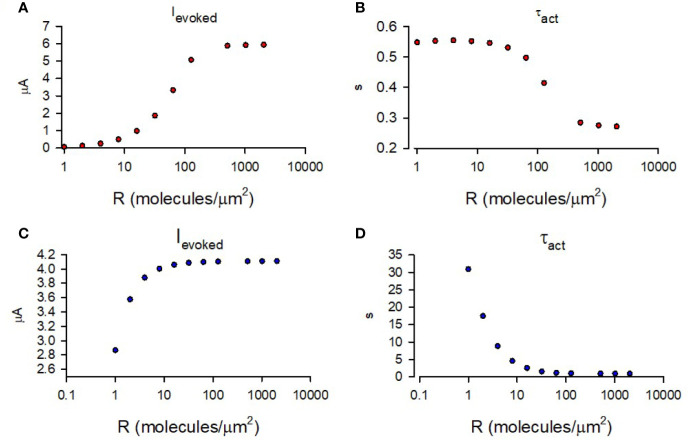
Simulation of GIRK1/2 activation according to cooperative gating model. **(A)** – Simulated values of I_evoked_ obtained for a range of M2R densities. **(B)** – τ_act_ of mono-exponential fit of simulated I_evoked_ obtained for a range of M2R densities. For A and B, the rate constants were taken from Touhara et al. (1) and it was assumed that Gβγ =16 molecules/channel and Gα = 9 molecules/channel (red circles). **(C)** – Simulated I_evoked_ obtained from simulation with k_hydrolysis_ = 0.02 s^-1^ and a range of M2R densities. **(D)** - τ_act_ of mono-exponential fit of time-course from simulation of I_evoked_ with k_hydrolysis_ = 0.02 s^-1^ and a range of M2R densities. For calculations done in **(C, D)**, k_hydrolysis_ was assumed to be 0.02 s^-1^ and Gβγ = 9 molecules/channel and Gα = 2 molecules/channel (blue circles).

Using the abovementioned initial conditions, we simulated the time-course of GIRK1/2 activation for a range of M2R densities similar to as shown in **Figure 4E**. Similar to data shown in **Figure 3** (for M2R-CFP), **Figures 1B** and **4E**, the amplitude of the simulated I_evoked_ increased with receptor density,whereas τ_act_ showed persistent decrease with the growing densities. However, the τ_act_ obtained was much faster than that observed in our experiments or predicted by our model, and varied less (i.e., of narrow range; **Figure 6B**). In order to determine the nature of this discrepancy, we compared similar kinetic steps between our model and that of cooperative gating model. We noticed that the GTP hydrolysis rate used by Touhara et al. is 100 times faster that used in other reports (Zhong et al., 2003). We also note that this fast rate of GTP hydrolysis is characteristic for conditions in which RGS is present, such as observed in cardiac myocytes (Doupnik, 2015). In our case, *Xenopus* oocytes lack RGS and thus slower GTP hydrolysis rates are expected (Keren-Raifman et al., 2001). Indeed, substituting slower hydrolysis rate constant into cooperative gating model restored channel activation kinetics to as obtained in our experiments (**Figures 6C, D**).

## Discussion

### Summary of Findings

The main goal of this work was to develop a detailed kinetic model for the GPCR-G-protein-effector cascade based on experimental data obtained in a prototypical expression system, the *Xenopus* oocyte. As many key features for this cascade are still missing, we deemed it quintessential to measure reactant densities in the plasma membrane along a detailed description of current features (amplitudes and kinetics). We used fluorescently labeled GIRK1/2 as a ‘molecular ruler” (Yakubovich et al., 2015) and conducted a controlled and quantitatively monitored expression of the component proteins of the signaling cascade, the GPCR (M2R) and the effector (GIRK1/2). G-protein concentrations were measured in a previous work using two independent methods, the fluorescence measurements with YFP-GIRK1/2 as caliper, and by quantitative Western blots (Yakubovich et al., 2015). Using a titrated expression approach (as previously developed by us; (Vorobiov et al., 2000)), we demonstrate that incremental expression of both wild-type and CFP-tagged M2R, M2R-CFP, is accompanied by acceleration (decrease in τ_act_) of channel activation, i.e., I_evoked_. In contrast, activation kinetics of GIRK responses elicited by an M2R-Gα_i3_ tandem, which necessarily mimics the case of a “preformed GPCR-G protein complex”, were fast and invariable over a wide range of expression levels of the fusion protein. We combined these observations and data to develop a comprehensive mathematical model for the M2R-G-protein-GIRK1/2 signaling cascade with free M2R based on free diffusion of all components, and a modification of this model for the case of M2R-Gα_i_ “preformed complex”. Our model faithfully recapitulates and predicts all the quantitative aspects of GIRK1/2 activation explored here: the acceleration of activation with increasing densities of M2R, the τ_act_ and the amplitude of I_evoked_, and the dependence of the amplitude, but not the kinetics, of GIRK currents elicited *via* the activation of the M2R-Gα_i3_ fusion protein. Our results strongly support the collision coupling mode of signaling between M2R and the G_i/o_ protein in the GPCR-G protein-GIRK cascade reconstituted in *Xenopus* oocytes. More broadly, our model, and a similar collision coupling type of model of Touhara et al. demonstrate that a purely diffusion-limited coupling mechanism can fully account for the fast kinetics of GIRK responses in excitable cells, without the need to assume a preformed complex. Importantly, our results emphasize the utility of our approach of controlled incremental GPCR expression to distinguish between different coupling modes in G protein-mediated signaling cascades.

### Model of G Protein Cycle

The mathematical approach used to describe the GPCR-G-protein-effector cascade is well elaborated (Lamb and Pugh, 1992; Turcotte et al., 2008; Falkenburger et al., 2010). Several models have been developed specifically for the analysis of GIRK activation. The first model of GIRK activation that also incorporated a G protein cycle of GIRK activation was published in 1988 (Breitwieser and Szabo, 1988). Subsequently, updated models have been developed by groups of Kurachi and Mackinnon (Murakami et al., 2010; Murakami et al., 2013; Touhara and MacKinnon, 2018). Though all these models implement Thomsen-Neubig style G-protein activation model (Thomsen and Neubig, 1989; Zhong et al., 2003), they differ in certain key details. In particular, the model proposed by Murakami et al. (2013) assumes two affinity states of GIRK to Gβγ and two affinity states of GPCR to agonist; however, the GPCR-agonist affinity states are not kinetically interconnected and unrelated to coupling to G-protein. These are incompatible with the well-established dependency of agonist affinity upon the G protein-GPCR association (Maguire et al., 1976; De Lean et al., 1980; Haga et al., 1986; Weis and Kobilka, 2018). The model proposed by Touhara and MacKinnon (2018) does not include an explicit bimolecular reaction of agonist binding to GPCR. Both models describe Gα_GTP_Gβγ heterotrimeric complex dissociation as an irreversible reaction, which precludes the implementation of the microscopic reversibility principle (Colquhoun et al., 2004).

The G-protein cycle model presented here (**Figure 4A**) is a logical development and, in a way, a synthesis of previously proposed models with certain improvements. In particular, receptor-agonist-G-protein interaction is formulated as a complete ternary complex model (De Lean et al., 1980). Both Gα_GTP_-Gβγ and Gα_GDP_-Gβγ interactions are described as reversible reactions. To enable the implementation of microscopic reversibility in the cycle, we have excluded the obligatorily irreversible GTP hydrolysis (**Figure 4A**, reaction 9) from the main cycle. Including only reversible reactions in the circular parts of G-protein activation model makes the model more thermodynamically plausible. Moreover, we do not assume that the proteins in the cascade are in unlimited supply, and the equations of the model completely take into account “depletion” of proteins by excess of their binding counterparts. The only exception is GTP and GDP that are assumed to be in unlimited supply (“free ligand approximation”, e.g. as is done for agonist in standard descriptions of agonist-receptor interactions).

Combining G-protein cycle model with GIRK1/2 gating model successfully reproduced the experimental observations. In particular, simulation of M2R*wt* expression experiment demonstrated similar decremental τ_act_ dependence on receptor density (**Figure 4**), a feature that was subsequently nearly abolished when modeling the M2R-Gα tandem activation process (**Figure 5**). These findings strengthen the notion that, in *Xenopus leaves* oocytes, M2R and G-proteins are not in “preformed complex”, rather interact reversibly.

### Channel Activation Models

The process that leads to GIRK opening following the binding of Gβγ is still unclear (Glaaser and Slesinger, 2017). Currently there are three detailed GIRK gating models developed chronologically by Kurachi group (Hosoya et al., 1996; Murakami et al., 2010; Murakami et al., 2013), our group (Yakubovich et al., 2005; Yakubovich et al., 2015) and MacKinnon’s group (Touhara et al., 2016; Wang et al., 2016; Touhara and MacKinnon, 2018). The model proposed by Kurachi’s group is based on Monod-Wyman-Changeux allosteric model of GIRK activation and formulates two binding states of Gβγ for each channel subunit. To complete the model, the authors used sub-nanomolar affinity for the GIRK-Gβγ interaction. The model developed by MacKinnon’s group includes a detailed binding reaction of Gβγ to GIRK2 and GIRK1/4 and is based on elegant experiments with purified GIRK2 and Gβγ incorporated into bilayer membranes. In their work, they suggest that Gβγ binding is cooperative and that only channels occupied by four Gβγ undergo the closed-open transition (Wang et al., 2016). The model presented in the current work is based on sequential binding of Gβγ molecules to GIRK1/2 with graded contribution of each Gβγ-occupied state to open probability. Notably, this is based on ample experiments using the homologous heterotetrameric GIRK1/4 (Ivanova-Nikolova and Breitwieser, 1997; Ivanova-Nikolova et al., 1998; Sadja et al., 2002). Considering the different affinity values, reported in the literature, for GIRK-Gβγ interaction (see below), we derived the K_D_ values of this interaction from analyzing the available crystal structure of GIRK2-Gβγ (Whorton and MacKinnon, 2013) and the docking model of GIRK1-Gβγ (Mahajan et al., 2013). Notably, both estimates rendered K_D_ values (100-200 nM) on par with those measured in biochemical experiments, 100-800 nM (Krapivinsky et al., 1995; Doupnik et al., 1996). Furthermore, our model of channel activation, combined with the G protein cycle model, reliably reproduced the amplitudes of GIRK1/2 activations when we used the surface densities of the channel, GPCR and G-protein directly measured under physiological conditions. We note that, with an appropriate adjustment for the oocyte expression system, the cooperative gating model of Touhara and MacKinnon (2018) also reproduced the experimental data reported here, in terms of GIRK1/2 currents, τ_act_ and the dependence of τ_act_ on M2R density (**Figure 6**). Interestingly, simulations done with the original parameters of the G-protein cycle model of Touhara et al. initially yielded a relatively shallow dependence of τ_act_ on M2R density, despite the fact that it is a collision coupling-type model. Analysis of this discrepancy lead us to the finding that τ_act_ dependence on receptor density even in “collision-coupling model” can be masked by a rapid rate of GTP hydrolysis, as can be obtained when RGS proteins are present (**Figure 6**).

A more consistent discrepancy of this model’s estimate with our estimates is related to estimations of the number of Gβγ and Gα molecules required for channel activation. The endogenous levels of Gβγ in oocyte’s plasma membrane is in the range of 20-40 molecules/µm^2^, and is further increased to ~80 molecules/µm^2^ (Yakubovich et al., 2015) upon overexpression of GIRK1/2 that recruits additional Gβγ to the plasma membrane (Rishal et al., 2005; Kahanovitch et al., 2014). Calculations with the Touhara et al. model showed that a minimum of 7 free Gβγ molecules need to be available at any time to account for the basal, GPCR-independent GIRK1/2 activity; at least 9 Gβγ and 2 Gα are needed to account for the observed I_total_ (I_basal_+I_evoked_) (**Figure S3**). For 21 GIRK1/2 channel/µm^2^ (as observed after injection of 1 ng RNA of each subunit; (Yakubovich et al., 2015)), ~75 Gβγ molecules per m^2^ are required to attain the total GIRK1/2 current according to our model, but ~180 Gβγ molecules per m^2^ are needed with the cooperative gating model. The differences in Gβγ estimates stem mainly from the distinct GIRK-Gβγ affinity estimates used: 60 µM for the first Gβγ bound, with progressively improved affinity for each following Gβγ in Touhara and MacKinnon (2018), vs. ~0.15 µM in our model (**Table 1**). An even greater discrepancy may be expected if one uses the K_D_ of ~300 µM for GIRK2-first Gβγ interaction, as estimated experimentally in lipid bilayer in the presence of Na^+^ (the natural condition in a living cell’s cytosol) (Wang et al., 2016). Touhara and MacKinnon (2018) also note that, with such low affinity of interaction between GIRK and Gβγ, Gβγ surface densities needed for GIRK activation should be much higher than the physiological range. This leads them to propose that the GPCRs, G-proteins and GIRK channels interact in hot spots (Sungkaworn et al., 2017), where all components of the cascade are highly concentrated. Another possible explanation is that the actual affinity of GIRK-Gβγ interaction is higher than 200-300 µM K_D_ despite the measurements in lipid bilayer experiments (Wang et al., 2016) or in solution by nuclear magnetic resonance (Yokogawa et al., 2011). Notably, these studies employed a mutant Gγ lacking lipid modification (geranylgeranylation, in the case of Gγ_2_) and it is established that lipid modification of Gγ is an important determinant of high-affinity interaction between Gβγ and its binding partners such as Gα and phosducin (Myung et al., 1999; Lukov et al., 2004). Further study is needed to better determine the affinity of GIRK-Gβγ interactions in living cells.

### Collision-Coupling Versus Preformed Complex

There is a longstanding debate regarding the existence, or lack, of diffusion-dependent steps in the GPCR-G-protein cycle. A large body of data suggests pre-formed (or dynamic) complexes between these proteins. For instance, measurement of diffusion coefficients of GPCRs demonstrate non-homogeneity, pointing to partial restriction of diffusion and possible organization of GPCRs in “islands” (Daumas et al., 2003; Suzuki et al., 2005; Baker et al., 2007). Similar restriction in lateral mobility is noted for G protein (Kwon et al., 1994). These observations are supported by studies showing that immobilized GPCRs can activate G-protein molecules with the same rate as mobile GPCRs (Lober et al., 2006). Going downstream in the cascade, the dissociation of Gα from Gβγ during the activation process also remains questioned. Whereas the dogma states full dissociation and diffusion of the latter, it has been shown that some Gα and Gβγ may not undergo dissociation, rather undergo spatial rearrangement after activation (Bunemann et al., 2003; Lambert, 2008). Onwards, complexes between G-protein and effectors, such as GIRK, have been noted (Robitaille et al., 2009; Zylbergold et al., 2010) as well as even larger supramolecular complexes consisting of GPCRs, G-proteins and modulating molecules, such as RGS, have been demonstrated (Lavine et al., 2002; Doupnik, 2008).Despite this body of work, pure collision coupling has also been demonstrated in many other cases (see introduction). Notably, the distinction between the two different modes is not trivial. In the current study, we elaborate our protocol using “titration” of proteins densities at the membrane, specifically those of the GPCR (Vorobiov et al., 2000) and show that it allows to quantitatively distinguish between the modes of GPCR-G protein coupling.

We compared the kinetic properties of two different settings of M2R-G-protein interaction. The first consists of M2R-Gα fusion protein; a one-to-one relationship between the GPCR and Gα is enforced, giving rise to a *bona fide* “preformed complex”. Notably, in the scenario, we also assume that the complex necessarily includes Gα_GDP_-Gβγ (in view of their very high nanomolar affinity), resulting in a full GPCR-G protein “preformed” complex. The second setting involves independent, untethered proteins. These two scenarios reveal that, whereas the kinetics of activation of GIRK by M2R*wt* are highly dependent on the receptor density, those of M2R-Gα fusion are not. This clear distinction strengthens the idea that, at least in *Xenopus* oocytes, M2R*wt* can indeed diffuse in the plasma membrane to activate several G-protein molecules (and GIRK subsequently). This conclusion is supported by studies, specifically conducted in *Xenopus* oocytes, demonstrating that, M2Rs and G-proteins are not permanently co-localized and diffuse unrestrictedly in the plasma membrane (Hein and Bunemann, 2009).

In summary, the current concept of GPCR-G-protein effector signaling may be schematically presented as three possible, and not mutually exclusive, paradigms. The first, the preformed complex model, is expected to follow first order kinetics, in which the rate of activation is concentration-independent. In the second, the catalytic collision coupling model, the rate of activation is anticipated to be highly concentration-dependent, at least for diffusion-limited cases. Of note, the dependence of activation kinetics on receptor density might also be influenced by G-protein inactivation rate. Indeed, we demonstrate this by employing the simulation of Touhara et al. with the slower GDP hydrolysis rate (**Figure 6**). The third, is a mixture of the two. Importantly, the relationship of activation rate and concentration of the reactants is not trivial and is formulated, in most cases, as differential rate laws rather than as integrated rate law utilized for first order kinetic processes (useful for preformed complexes). It was previously shown that coupling reaction rate constant under diffusion limited conditions is equal to diffusion transport constant (Lauffenburger and Linderman, 1996) and is therefore expected to be dependent on receptor density (Mahama and Linderman, 1994; Shea et al., 1997). Together, the dependence of GIRK’s activation rate on the density of M2R most likely originates from this phenomenon as a result of collision coupling of GPCR and G-protein. The recently proposed “hot spot” interaction model of GPCR-G-protein activation (Suzuki et al., 2005; Sungkaworn et al., 2017) represents a particular case of collision coupling model (but not preformed complexes) and elegantly describes cases in which there is relatively low affinity between the reactants, because restrictions of molecules within a tight hot spot is expected to robustly increase their effective concentration. Further studies based on stochastic analysis of GPCR-G-protein-GIRK system and measurement of GIRK-Gβγ affinity will deepen our understanding of the above described phenomena.

## Data Availability Statement

The raw data supporting the conclusions of this article will be made available by the authors, without undue reservation.

## Ethics Statement

The animal study was reviewed and approved by Tel Aviv University Institutional Animal Care and Use Committee (permits M-08-081 and M-13-002).

## Author Contributions

SB, EA, RH-J, UK—electrophysiological and imaging experiments. DY, HP—generation of the model. ND, SB, DY—planning of the study. DY, ND, SB—writing the first draft of the manuscript. All authors contributed to the article and approved the submitted version.

## Funding

This work was supported by the Israel Science Foundation (N.D.- grant #1282/18, and S.B.- grant #1096/17) and the Mauerberg Cathedra for Neuropharmacology (N.D.).

## Conflict of Interest

The authors declare that the research was conducted in the absence of any commercial or financial relationships that could be construed as a potential conflict of interest.

The handling editor declared a shared affiliation, though no other collaboration, with one of the authors HP.

## Supplementary Material

Supplemental Figure S1 Kinetic measurements of time course of AMPA-R activation. **(A)** –configuration of the experimental chamber used for fast perfusion experiments. **(B)**– a representative record (black dots) of current evoked by 1 mM glutamate. The red dots represent mono-exponential fit of the activation time course of the current (τ_act_=49.5 s).

Supplemental Figure S2 Linear relationship between Gβγ-activated YFP-GIRK1/GIRK2 current and total surface density is observed in the range 0-1 ng of YFP-GIRK1 RNA. **(A)** – representative confocal images of oocytes expressing YFP-GIRK1/GIRK2. Channels were expressed by injecting the indicated doses of RNA of YFP-GIRK1 and GIRK2 (1:1) and activated by coexpression of 5 ng Gβ and 1 ng Gγ RNA. **(B)** – correlation between total GIRK surface density and the Gβγ-dependent GIRK current, I_βγ_. I_βγ_ is the total agonist-independent current in Gβγ-expressing oocytes. It was measured in 24 mM K^+^ solution by subtracting the non-GIRK currents remaining after inhibition of >95% GIRK activity by 5 mM Ba^2+^ (Rubinstein et al., 2007). Total surface density is reflected in YFP fluorescence levels, functional channel density is proportional to I_βγ_. Correlation between I_βγ_ and fluorescence is linear for RNA doses of YFP-GIRK1/GIRK2 up to 1 ng/oocyte of each subunit. n = 6-12 for each point.

Supplemental Figure S3 Estimation of Gβγ and Gα densities for simulation with cooperative gating model. **(A)** – I_basal_ currents rendered by different pairs of Gβγ and Gα densities selected for simulation. **(B)** – I_total_ currents rendered by different pairs of Gβγ and Gα densities selected for simulation.Red bars indicate Gβγ and Gα densities used for calculations in **Figures 6A, B**. Blue bars indicate Gβγ and Gα densities used for calculations in **Figures 6C, D**. All calculations except the 9:2 Gβγ/Gα pair (blue bar) have been made for the condition of fast hydrolysis of GTP, k_hydrolysis_=2 s^-1^. The 16:9 Gβγ/Gα pair was the lowest dose of G protein subunits per channel that reproduced I_total_ under this condition. Lower doses of Gβγ/Gα produced lower I_total_. The 9:2 Gβγ/Gα pair was the lowest G protein subunits combination that reproduced I_total_ under the condition of low GTP hydrolysis rate (k_hydrolysis_=0.02 s^-1^).

## References

[B1] Alsallaq R. Zhou H. X. (2008). Electrostatic rate enhancement and transient complex of protein-protein association. Proteins 71, 320–335. 10.1002/prot.21679 17932929 PMC3526769

[B2] Arshavsky V. Y. Lamb T. D. Pugh E. N. Jr. (2002). G proteins and phototransduction. Annu. Rev. Physiol. 64, 153–187. 10.1146/annurev.physiol.64.082701.102229 11826267

[B3] Baker A. Sauliere A. Dumas F. Millot C. Mazeres S. Lopez A. (2007). Functional membrane diffusion of G-protein coupled receptors. Eur. Biophys. J. 36, 849–860. 10.1007/s00249-007-0214-7 17899063

[B4] Ben-Chaim Y. Tour O. Dascal N. Parnas I. Parnas H. (2003). The M2 muscarinic G-protein-coupled receptor is voltage sensitive. J. Biol. Chem. 278, 22482–22491. 10.1074/jbc.M301146200 12684524

[B5] Bender K. Wellner-Kienitz M. C. Meyer T. Pott L. (1998). Activation of muscarinic K^+^ current by b-adrenergic receptors in cultured atrial myocytes transfected with b1 subunit of heterotrimeric G proteins. FEBS Lett. 439, 115–120. 10.1016/S0014-5793(98)01350-7 9849890

[B6] Berlin S. Keren-Raifman T. Castel R. Rubinstein M. Dessauer C. W. Ivanina T. (2010). Gα_i_ and Gβγ jointly regulate the conformations of a Gβγ effector, the neuronal G protein-activated K^+^ channel (GIRK). J. Biol. Chem. 285, 6179–6185. 10.1074/jbc.M109.085944 20018875 PMC2825413

[B7] Berlin S. Tsemakhovich V. A. Castel R. Ivanina T. Dessauer C. W. Keren-Raifman T. (2011). Two distinct aspects of coupling between Gαi protein and G protein-activated K^+^ channel (GIRK) revealed by fluorescently labeled Gα_i3_ protein subunits. J. Biol. Chem. 286, 33223–33235. 10.1074/jbc.M111.271056 21795707 PMC3190912

[B8] Breitwieser G. E. Szabo G. (1988). Mechanism of Muscarinic Receptor Induced K+ Channel Activation as Revealed by Hydrolysis-Resistant Gtp Analogs. J. Gen. Physiol. 91, 469–493. 10.1085/jgp.91.4.469 2455765 PMC2216147

[B9] Bunemann M. Frank M. Lohse M. J. (2003). Gi protein activation in intact cells involves subunit rearrangement rather than dissociation. Proc. Natl. Acad. Sci. U. S. A. 100, 16077–16082. 10.1073/pnas.2536719100 14673086 PMC307695

[B10] Clapham D. E. Neer E. J. (1997). G protein βγ subunits. Annu. Rev. Pharmacol. Toxicol. 37, 167–203. 10.1146/annurev.pharmtox.37.1.167 9131251

[B11] Colquhoun D. Dowsland K. A. Beato M. Plested A. J. (2004). How to impose microscopic reversibility in complex reaction mechanisms. Biophys. J. 86, 3510–3518. 10.1529/biophysj.103.038679 15189850 PMC1304255

[B12] Corey S. Krapivinsky G. Krapivinsky L. Clapham D. E. (1998). Number and stoichiometry of subunits in the native atrial G-protein-gated K+ channel, IKACh. J. Biol. Chem. 273, 5271–5278. 10.1074/jbc.273.9.5271 9478984

[B13] Dascal N. Kahanovitch U. (2015). The roles of Gβγ and Gα in gating and regulation of GIRK channels. Int. Rev. Neurobiol. 123, 27–85. 10.1016/bs.irn.2015.06.001 26422982

[B14] Dascal N. Lotan I. (1992). “Expression of exogenous ion channels and neurotransmitter receptors in RNA-injected Xenopus oocytes,” in Protocols in Molecular Neurobiology. Eds. Longstaff A. Revest P. (Totowa, NJ: Humana Press), 205–225.

[B15] Dascal N. (1987). The use of Xenopus oocytes for the study of ion channels. CRC Crit. Rev. Biochem. 22, 317–387. 10.3109/10409238709086960 2449311

[B16] Dascal N. (1997). Signalling *via the* G protein-activated K^+^ channels. Cell Signal 9, 551–573. 10.1016/S0898-6568(97)00095-8 9429760

[B17] Daumas F. Destainville N. Millot C. Lopez A. Dean D. Salome L. (2003). Confined diffusion without fences of a g-protein-coupled receptor as revealed by single particle tracking. Biophys. J. 84, 356–366. 10.1016/S0006-3495(03)74856-5 12524289 PMC1302617

[B18] De Lean A. Stadel J. M. Lefkowitz R. J. (1980). A ternary complex model explains the agonist-specific binding properties of the adenylate cyclase-coupled β-adrenergic receptor. J. Biol. Chem. 255, 7108–7117. 6248546

[B19] Doupnik C. A. Dessauer C. W. Slepak V. Z. Gilman A. G. Davidson N. Lester H. A. (1996). Time resolved kinetics of direct Gb_1_g_2_ interactions with the carboxyl terminus of Kir3.4 inward rectifier K^+^ channel subunits. Neuropharmacology 35, 923–931. 10.1016/0028-3908(96)00125-6 8938723

[B20] Doupnik C. A. (2008). GPCR-Kir channel signaling complexes: defining rules of engagement. J. Recept Signal Transduct. Res. 28, 83–91. 10.1080/10799890801941970 18437632

[B21] Doupnik C. A. (2015). RGS redundancy and implications in GPCR-GIRK signaling. Int. Rev. Neurobiol. 123, 87–116. 10.1016/bs.irn.2015.05.010 26422983

[B22] Dupre D. J. Baragli A. Rebois R. V. Ethier N. Hebert T. E. (2007). Signalling complexes associated with adenylyl cyclase II are assembled during their biosynthesis. Cell Signal 19, 481–489. 10.1016/j.cellsig.2006.07.021 16979872

[B23] Dupre D. J. Robitaille M. Rebois R. V. Hebert T. E. (2009). The role of Gβγ subunits in the organization, assembly, and function of GPCR signaling complexes. Annu. Rev. Pharmacol. Toxicol. 49, 31–56. 10.1146/annurev-pharmtox-061008-103038 18834311 PMC2659589

[B24] Falkenburger B. H. Jensen J. B. Hille B. (2010). Kinetics of M1 muscarinic receptor and G protein signaling to phospholipase C in living cells. J. Gen. Physiol. 135, 81–97. 10.1085/jgp.200910344 20100890 PMC2812500

[B25] Fowler C. E. Aryal P. Suen K. F. Slesinger P. A. (2006). Evidence for association of GABA_B_ receptors with Kir3 channels and RGS4 proteins. J. Physiol. (Lond) 580, 51–65. 10.1113/jphysiol.2006.123216 17185339 PMC2075413

[B26] Glaaser I. W. Slesinger P. A. (2017). Dual activation of neuronal G protein-gated inwardly rectifying potassium (GIRK) channels by cholesterol and alcohol. Sci. Rep. 7, 4592. 10.1038/s41598-017-04681-x 28676630 PMC5496853

[B27] Grosskreutz J. Zoerner A. Schlesinger F. Krampfl K. Dengler R. Bufler J. (2003). Kinetic properties of human AMPA-type glutamate receptors expressed in HEK293 cells. Eur. J. Neurosci. 17, 1173–1178. 10.1046/j.1460-9568.2003.02531.x 12670305

[B28] Haga K. Haga T. Ichiyama A. (1986). Reconstitution of the muscarinic acetylcholine receptor. Guanine nucleotide-sensitive high affinity binding of agonists to purified muscarinic receptors reconstituted with GTP-binding proteins (Gi and Go). J. Biol. Chem. 261, 10133–10140. 3015919

[B29] Halvorsen S. W. Nathanson N. M. (1981). In vivo regulation of muscarinic acetylcholine receptor number and function in embryonic chick heart. J. Biol. Chem. 256, 7941–7948. 7263635

[B30] Hein P. Bunemann M. (2009). Coupling mode of receptors and G proteins. Naunyn Schmiedebergs Arch. Pharmacol. 379, 435–443. 10.1007/s00210-008-0383-7 19048232

[B31] Hein P. Frank M. Hoffmann C. Lohse M. J. Bunemann M. (2005). Dynamics of receptor/G protein coupling in living cells. EMBO J. 24, 4106–4114. 10.1038/sj.emboj.7600870 16292347 PMC1356310

[B32] Hibino H. Inanobe A. Furutani K. Murakami S. Findlay I. Kurachi Y. (2010). Inwardly rectifying potassium channels: their structure, function, and physiological roles. Physiol. Rev. 90, 291–366. 10.1152/physrev.00021.2009 20086079

[B33] Higashijima T. Ferguson K. M. Sternweis P. C. Smigel M. D. Gilman A. G. (1987). Effects of Mg2+ and the beta gamma-subunit complex on the interactions of guanine nucleotides with G proteins. J. Biol. Chem. 262, 762–766. 3100519

[B34] Hille B. Dickson E. Kruse M. Falkenburger B. (2014). Dynamic metabolic control of an ion channel. Prog. Mol. Biol. Transl. Sci. 123, 219–247. 10.1016/B978-0-12-397897-4.00008-5 24560147

[B35] Hille B. (1992). G protein-coupled mechanisms and nervous signaling. Neuron 9, 187–195. 10.1016/0896-6273(92)90158-A 1353972

[B36] Hille B. (2002). Ion Channels of Excitable Membranes (Sunderland: Sinauer).

[B37] Hollins B. Kuravi S. Digby G. J. Lambert N. A. (2009). The c-terminus of GRK3 indicates rapid dissociation of G protein heterotrimers. Cell Signal 21, 1015–1021. 10.1016/j.cellsig.2009.02.017 19258039 PMC2668204

[B38] Hosoya Y. Yamada M. Ito H. Kurachi Y. (1996). A functional model for G protein activation of the muscarinic K^+^ channel in guinea pig atrial myocytes. Spectral analysis of the effect of GTP on single-channel kinetics. J. Gen. Physiol. 108, 485–495. 10.1085/jgp.108.6.485 8972387 PMC2229342

[B39] Huang C. L. Slesinger P. A. Casey P. J. Jan Y. N. Jan L. Y. (1995). Evidence that direct binding of Gbg to the GIRK1 G protein- gated inwardly rectifying K^+^ channel is important for channel activation. Neuron 15, 1133–1143. 10.1016/0896-6273(95)90101-9 7576656

[B40] Ilyaskina O. S. Lemoine H. Bunemann M. (2018). Lifetime of muscarinic receptor-G-protein complexes determines coupling efficiency and G-protein subtype selectivity. Proc. Natl. Acad. Sci. U. S. A. 115, 5016–5021. 10.1073/pnas.1715751115 29686069 PMC5948956

[B41] Ivanova-Nikolova T. T. Breitwieser G. E. (1997). Effector contributions to Gbg-mediated signaling as revealed by muscarinic potassium channel gating. J. Gen. Physiol. 109, 245–253. 10.1085/jgp.109.2.245 9041452 PMC2220061

[B42] Ivanova-Nikolova T. T. Nikolov E. N. Hansen C. Robishaw J. D. (1998). Muscarinic K^+^ channel in the heart. Modal regulation by G protein bg subunits. J. Gen. Physiol. 112, 199–210. 10.1085/jgp.112.2.199 9689027 PMC2525744

[B43] Jaen C. Doupnik C. A. (2006). RGS3s and RGS4 differentially associate with GPCR-Kir3 channel signaling complexes revealing 2 modes of RGS modulation: precoupling and collision-coupling. J. Biol. Chem. 281, 34549–34560. 10.1074/jbc.M603177200 16973624

[B44] Kahanovitch U. Tsemakhovich V. Berlin S. Rubinstein M. Styr B. Castel R. (2014). Recruitment of Gβγ controls the basal activity of G-protein coupled inwardly rectifying potassium (GIRK) channels: crucial role of distal C terminus of GIRK1. J. Physiol. 592, 5373–5390. 10.1113/jphysiol.2014.283218 25384780 PMC4270501

[B45] Kahanovitch U. Berlin S. Dascal N. (2017). Collision coupling in the GABA_B_ receptor - G protein - GIRK signaling cascade. FEBS Lett. 591, 2816–2825. 10.1002/1873-3468.12756 28724189

[B46] Kano H. Toyama Y. Imai S. Iwahashi Y. Mase Y. Yokogawa M. (2019). Structural mechanism underlying G protein family-specific regulation of G protein-gated inwardly rectifying potassium channel. Nat. Commun. 10, 2008. 10.1038/s41467-019-10038-x 31043612 PMC6494913

[B47] Kastritis P. L. Moal I. H. Hwang H. Weng Z. Bates P. A. Bonvin A. M. (2011). A structure-based benchmark for protein-protein binding affinity. Protein Sci. 20, 482–491. 10.1002/pro.580 21213247 PMC3064828

[B48] Keren-Raifman T. Bera A. K. Zveig D. Peleg S. Witherow D. S. Slepak V. Z. (2001). Expression levels of RGS7 and RGS4 proteins determine the mode of regulation of the G protein-activated K^+^ channel and control regulation of RGS7 by Gb5. FEBS Lett. 492, 20–28. 10.1016/S0014-5793(01)02220-7 11248230

[B49] Krapivinsky G. Krapivinsky L. Wickman K. Clapham D. E. (1995). Gbg binds directly to the G protein-gated K^+^ channel, I_KACh_. J. Biol. Chem. 270, 29059–29062. 10.1074/jbc.270.49.29059 7493925

[B50] Kwon G. Axelrod D. Neubig R. R. (1994). Lateral mobility of tetramethylrhodamine (TMR) labelled G protein α and β γ subunits in NG 108-15 cells. Cell Signal 6, 663–679. 10.1016/0898-6568(94)90049-3 7857770

[B51] Lamb T. D. Pugh E. N. Jr. (1992). G-protein cascades: gain and kinetics. Trends Neurosci. 15, 291–298. 10.1016/0166-2236(92)90079-N 1384198

[B52] Lambert N. A. (2008). Dissociation of heterotrimeric g proteins in cells. Sci. Signal 1, re5. 10.1126/scisignal.125re5 18577758

[B53] Lauffenburger D. Linderman J. J. (1996). Receptors: Models for Binding, Trafficking, and Signaling (New York: Oxford University Press).

[B54] Lavine N. Ethier N. Oak J. N. Pei L. Liu F. Trieu P. (2002). G protein-coupled receptors form stable complexes with inwardly rectifying potassium channels and adenylyl cyclase. J. Biol. Chem. 277, 46010–46019. 10.1074/jbc.M205035200 12297500

[B55] Lechleiter J. Hellmiss R. Duerson K. Ennulat D. David N. Clapham D. (1990). Distinct sequence elements control the specificity of G protein activation by muscarinic acetylcholine receptor subtypes. EMBO J. 9, 4381–4390. 10.1002/j.1460-2075.1990.tb07888.x 2124972 PMC552228

[B56] Lim N. F. Dascal N. Labarca C. Davidson N. Lester H. A. (1995). A G protein-gated K channel is activated *via* b2-adrenergic receptors and Gbg subunits in *Xenopus* oocytes. J. Gen. Physiol. 105, 421–439. 10.1085/jgp.105.3.421 7769382 PMC2216943

[B57] Liman E. R. Tytgat J. Hess P. (1992). Subunit stoichiometry of a mammalian K^+^ channel determined by construction of multimeric cDNAs. Neuron 9, 861–871. 10.1016/0896-6273(92)90239-A 1419000

[B58] Lober R. M. Pereira M. A. Lambert N. A. (2006). Rapid activation of inwardly rectifying potassium channels by immobile G-protein-coupled receptors. J. Neurosci. 26, 12602–12608. 10.1523/JNEUROSCI.4020-06.2006 17135422 PMC6674890

[B59] Logothetis D. E. Kurachi Y. Galper J. Neer E. J. Clapham D. E. (1987). The βγ subunits of GTP-binding proteins activate the muscarinic K+ channel in heart. Nature 325, 321–326. 10.1038/325321a0 2433589

[B60] Lohse M. J. Hein P. Hoffmann C. Nikolaev V. O. Vilardaga J. P. Bunemann M. (2008). Kinetics of G-protein-coupled receptor signals in intact cells. Br. J. Pharmacol. 153 Suppl 1, S125–S132. 10.1038/sj.bjp.0707656 18193071 PMC2268076

[B61] Lopatin A. N. Makhina E. N. Nichols C. G. (1998). A novel crystallization method for visualizing the membrane localization of potassium channels. Biophys. J. 74, 2159–2170. 10.1016/S0006-3495(98)77925-1 9591643 PMC1299559

[B62] Lukov G. L. Myung C. S. Mcintire W. E. Shao J. Zimmerman S. S. Garrison J. C. (2004). Role of the isoprenyl pocket of the G protein β γ subunit complex in the binding of phosducin and phosducin-like protein. Biochemistry 43, 5651–5660. 10.1021/bi035903u 15134439

[B63] Luscher C. Slesinger P. A. (2010). Emerging roles for G protein-gated inwardly rectifying potassium (GIRK) channels in health and disease. Nat. Rev. Neurosci. 11, 301–315. 10.1038/nrn2834 20389305 PMC3052907

[B64] Machaca K. Hartzell H. C. (1998). Asymmetrical distribution of Ca-activated Cl channels in Xenopus oocytes. Biophys. J. 74, 1286–1295. 10.1016/S0006-3495(98)77842-7 9512026 PMC1299476

[B65] Maeda S. Qu Q. Robertson M. J. Skiniotis G. Kobilka B. K. (2019). Structures of the M1 and M2 muscarinic acetylcholine receptor/G-protein complexes. Science 364, 552–557. 10.1126/science.aaw5188 31073061 PMC7034192

[B66] Maguire M. E. Van Arsdale P. M. Gilman A. G. (1976). An agonist-specific effect of guanine nucleotides on binding to the β-adrenergic receptor. Mol. Pharmacol. 12, 335–339. 4726

[B67] Mahajan R. Ha J. Zhang M. Kawano T. Kozasa T. Logothetis D. E. (2013). A computational model predicts that Gβγ acts at a cleft between channel subunits to activate GIRK1 channels. Sci. Signal 6, ra69. 10.1126/scisignal.2004075 23943609 PMC4100999

[B68] Mahama P. A. Linderman J. J. (1994). A Monte Carlo study of the dynamics of G-protein activation. Biophys. J. 67, 1345–1357. 10.1016/S0006-3495(94)80606-X 7811949 PMC1225491

[B69] Mayfield J. Blednov Y. A. Harris R. A. (2015). Behavioral and genetic evidence for GIRK channels in the CNS: role in physiology, pathophysiology, and drug addiction. Int. Rev. Neurobiol. 123, 279–313. 10.1016/bs.irn.2015.05.016 26422988 PMC4769645

[B70] Murakami S. Suzuki S. Ishii M. Inanobe A. Kurachi Y. (2010). Cellular modelling: experiments and simulation to develop a physiological model of G-protein control of muscarinic K^+^ channels in mammalian atrial cells. Philos. Trans. A Math Phys. Eng. Sci. 368, 2983–3000. 10.1098/rsta.2010.0093 20478917

[B71] Murakami S. Inanobe A. Kurachi Y. (2013). Short-term desensitization of muscarinic K^+^ current in the heart. Biophys. J. 105, 1515–1525. 10.1016/j.bpj.2013.08.009 24048003 PMC3785892

[B72] Myung C. S. Yasuda H. Liu W. W. Harden T. K. Garrison J. C. (1999). Role of isoprenoid lipids on the heterotrimeric G protein γ subunit in determining effector activation. J. Biol. Chem. 274, 16595–16603. 10.1074/jbc.274.23.16595 10347226

[B73] Nagi K. Pineyro G. (2014). Kir3 channel signaling complexes: focus on opioid receptor signaling. Front. Cell Neurosci. 8, 186. 10.3389/fncel.2014.00186 25071446 PMC4085882

[B74] Pott L. (1979). On the time course of the acetylcholine-induced hyperpolarization in quiescent guinea-pig atria. Pflugers Arch. 380, 71–77. 10.1007/BF00582615 572042

[B75] Qin S. Pang X. Zhou H. X. (2011). Automated prediction of protein association rate constants. Structure 19, 1744–1751. 10.1016/j.str.2011.10.015 22153497 PMC3240845

[B76] Rebois R. V. Robitaille M. Gales C. Dupre D. J. Baragli A. Trieu P. (2006). Heterotrimeric G proteins form stable complexes with adenylyl cyclase and Kir3.1 channels in living cells. J. Cell Sci. 119, 2807–2818. 10.1242/jcs.03021 16787947

[B77] Reuveny E. Slesinger P. A. Inglese J. Morales J. M. Iniguez-Lluhi J. A. Lefkowitz R. J. (1994). Activation of the cloned muscarinic potassium channel by G protein bg subunits. Nature 370, 143–146. 10.1038/370143a0 8022483

[B78] Richter J. D. Smith L. D. (1981). Differential capacity for translation and lack of competition between mRNAs that segregate to free and membrane-bound polysomes. Cell 27, 183–191. 10.1016/0092-8674(81)90372-X 7198941

[B79] Rifkin R. A. Moss S. J. Slesinger P. A. (2017). G protein-gated potassium channels: A link to drug addiction. Trends Pharmacol. Sci. 38, 378–392. 10.1016/j.tips.2017.01.007 28188005 PMC5368012

[B80] Rishal I. Porozov Y. Yakubovich D. Varon D. Dascal N. (2005). Gβγ-dependent and Gβγ-independent basal activity of G protein-activated K^+^ channels. J. Biol. Chem. 280, 16685–16694. 10.1074/jbc.M412196200 15728579

[B81] Riven I. Iwanir S. Reuveny E. (2006). GIRK channel activation involves a local rearrangement of a preformed G protein channel complex. Neuron 51, 561–573. 10.1016/j.neuron.2006.08.017 16950155

[B82] Robinson K. R. (1979). Electrical currents through full-grown and maturing Xenopus oocytes. Proc. Natl. Acad. Sci. U. S. A. 76, 837–841. 10.1073/pnas.76.2.837 284407 PMC383067

[B83] Robitaille M. Ramakrishnan N. Baragli A. Hebert T. E. (2009). Intracellular trafficking and assembly of specific Kir3 channel/G protein complexes. Cell Signal 21, 488–501. 10.1016/j.cellsig.2008.11.011 19135528

[B84] Ross E. M. (2008). Coordinating speed and amplitude in G-protein signaling. Curr. Biol. 18, R777–R783. 10.1016/j.cub.2008.11.032 18786383 PMC2654212

[B85] Rubinstein M. Peleg S. Berlin S. Brass D. Dascal N. (2007). Ga_i3_ primes the G protein-activated K^+^ channels for activation by coexpressed Gbg in intact *Xenopus* oocytes. J. Physiol. 581, 17–32. 10.1113/jphysiol.2006.125864 17289785 PMC2075207

[B86] Rubinstein M. Peleg S. Berlin S. Brass D. Keren-Raifman T. Dessauer C. W. (2009). Divergent regulation of GIRK1 and GIRK2 subunits of the neuronal G protein gated K^+^ channel by Ga_i_GDP and Gbg. J. Physiol. 587, 3473–3491. 10.1113/jphysiol.2009.173229 19470775 PMC2742276

[B87] Rusinova R. Mirshahi T. Logothetis D. E. (2007). Specificity of Gβγ signaling to Kir3 channels depends on the helical domain of pertussis toxin-sensitive Galpha subunits. J. Biol. Chem. 282, 34019–34030. 10.1074/jbc.M704928200 17872944

[B88] Sadja R. Alagem N. Reuveny E. (2002). Graded contribution of the Gbg binding domains to GIRK channel activation. Proc. Natl. Acad. Sci. U. S. A. 99, 10783–10788. 10.1073/pnas.162346199 12124401 PMC125044

[B89] Sarvazyan N. A. Remmers A. E. Neubig R. R. (1998). Determinants of G_i1_a and bg binding. Measuring high affinity interactions in a lipid environment using flow cytometry. J. Biol. Chem. 273, 7934–7940. 10.1074/jbc.273.14.7934 9525890

[B90] Sarvazyan N. A. Lim W. K. Neubig R. R. (2002). Fluorescence analysis of receptor-G protein interactions in cell membranes. Biochemistry 41, 12858–12867. 10.1021/bi026212l 12379129

[B91] Schreiber G. Henis Y. I. Sokolovsky M. (1985). Rate constants of agonist binding to muscarinic receptors in rat brain medulla. Evaluation by competition kinetics. J. Biol. Chem. 260, 8795–8802. 4019454

[B92] Schreibmayer W. Lester H. A. Dascal N. (1994). Voltage clamping of *Xenopus laevis* oocytes utilizing agarose-cushion electrodes. Pflugers Arch. Eur. J. Physiol. 426, 453–458. 10.1007/BF00388310 7517034

[B93] Shea L. Linderman J. J. (1997). Mechanistic model of G-protein signal transduction. Determinants of efficacy and effect of precoupled receptors. Biochem. Pharmacol. 53, 519–530. 10.1016/S0006-2952(96)00768-X 9105403

[B94] Shea L. D. Omann G. M. Linderman J. J. (1997). Calculation of diffusion-limited kinetics for the reactions in collision coupling and receptor cross-linking. Biophys. J. 73, 2949–2959. 10.1016/S0006-3495(97)78323-1 9414209 PMC1181200

[B95] Shea L. D. Neubig R. R. Linderman J. J. (2000). Timing is everything - The role of kinetics in G protein activation. Life Sci. 68, 647–658. 10.1016/S0024-3205(00)00977-2 11205879

[B96] Silverman S. K. Lester H. A. Dougherty D. A. (1996). Subunit stoichiometry of a heteromultimeric G protein-coupled inward-rectifier K+ channel. J. Biol. Chem. 271, 30524–30528. 10.1074/jbc.271.48.30524 8940021

[B97] Slesinger P. A. Reuveny E. Jan Y. N. Jan L. Y. (1995). Identification of structural elements involved in G protein gating of the GIRK1 potassium channel. Neuron 15, 1145–1156. 10.1016/0896-6273(95)90102-7 7576657

[B98] Sodickson D. L. Bean B. P. (1996). GABA_B_ receptor-activated inwardly rectifying potassium current in dissociated hippocampal CA3 neurons. J. Neurosci. 16, 6374–6385. 10.1523/JNEUROSCI.16-20-06374.1996 8815916 PMC6578909

[B99] Stern-Bach Y. Russo S. Neuman M. Rosenmund C. (1998). A point mutation in the glutamate binding site blocks desensitization of AMPA receptors. Neuron 21, 907–918. 10.1016/S0896-6273(00)80605-4 9808475

[B100] Sungkaworn T. Jobin M.-L. Burnecki K. Weron A. Lohse M. J. Calebiro D. (2017). Single-molecule imaging reveals receptor–G protein interactions at cell surface hot spots. Nature 550, 543–547. 10.1038/nature24264 29045395

[B101] Suzuki K. Ritchie K. Kajikawa E. Fujiwara T. Kusumi A. (2005). Rapid hop diffusion of a G-protein-coupled receptor in the plasma membrane as revealed by single-molecule techniques. Biophys. J. 88, 3659–3680. 10.1529/biophysj.104.048538 15681644 PMC1305513

[B102] Tabak G. Keren-Raifman T. Kahanovitch U. Dascal N. (2019). Mutual action by Gγ and Gβ for optimal activation of GIRK channels in a channel subunit-specific manner. Sci. Rep. 9, 508. 10.1038/s41598-018-36833-y 30679535 PMC6346094

[B103] Tateyama M. Kubo Y. (2018). Gi/o-coupled muscarinic receptors co-localize with GIRK channel for efficient channel activation. PLoS One 13, e0204447. 10.1371/journal.pone.0204447 30240440 PMC6150519

[B104] Thomsen W. J. Neubig R. R. (1989). Rapid kinetics of α 2-adrenergic inhibition of adenylate cyclase. Evidence for a distal rate-limiting step. Biochemistry 28, 8778–8786. 10.1021/bi00448a015 2574993

[B105] Thomsen W. J. Jacquez J. A. Neubig R. R. (1988). Inhibition of adenylate cyclase is mediated by the high affinity conformation of the α 2-adrenergic receptor. Mol. Pharmacol. 34, 814–822. 2904647

[B106] Tolkovsky A. M. Levitzki A. (1981). Theories and predictions of models describing sequential interactions between the receptor, the GTP regulatory unit, and the catalytic unit of hormone dependent adenylate cyclases. J. Cyclic Res. 7, 139–150. 6270199

[B107] Tolkovsky A. M. Braun S. Levitzki A. (1982). Kinetics of Interaction between β-Receptors, Gtp Protein, and the Catalytic Unit of Turkey Erythrocyte Adenylate-Cyclase. Proc. Natl. Acad. Sci. U. States America 79, 213–217. 10.1073/pnas.79.2.213 PMC345696 6281756

[B108] Touhara K. K. MacKinnon R. (2018). Molecular basis of signaling specificity between GIRK channels and GPCRs. eLife 7, e42908. 10.7554/eLife.42908 30526853 PMC6335053

[B109] Touhara K. K. Wang W. Mackinnon R. (2016). The GIRK1 subunit potentiates G protein activation of cardiac GIRK1/4 hetero-tetramers. Elife 5, e15750. 10.7554/eLife.15750 27074664 PMC4866825

[B110] Traut T. W. (1994). Physiological concentrations of purines and pyrimidines. Mol. Cell Biochem. 140, 1–22. 10.1007/BF00928361 7877593

[B111] Turcotte M. Tang W. Ross E. M. (2008). Coordinate regulation of G protein signaling *via* dynamic interactions of receptor and GAP. PLoS Comput. Biol. 4, e1000148. 10.1371/journal.pcbi.1000148 18716678 PMC2518520

[B112] Voigt N. Abu-Taha I. Heijman J. Dobrev D. (2014). Constitutive activity of the acetylcholine-activated potassium current I_K_,_ACh_ in cardiomyocytes. Adv. Pharmacol. 70, 393–409. 10.1016/B978-0-12-417197-8.00013-4 24931203

[B113] Vorobiov D. Bera A. K. Keren-Raifman T. Barzilai R. Dascal N. (2000). Coupling of the muscarinic m2 receptor to G protein-activated K^+^ channels *via* Ga_z_ and a receptor-Ga_z_ fusion protein. Fusion between the receptor and Ga_z_ eliminates catalytic (collision) coupling. J. Biol. Chem. 275, 4166–4170. 10.1074/jbc.275.6.4166 10660578

[B114] Wang W. Touhara K. K. Weir K. Bean B. P. Mackinnon R. (2016). Cooperative regulation by G proteins and Na^+^ of neuronal GIRK2 K^+^ channels. Elife 5, e15751. 10.7554/eLife.15751 27074662 PMC4866826

[B115] Weis W. I. Kobilka B. K. (2018). The Molecular Basis of G Protein-Coupled Receptor Activation. Annu. Rev. Biochem. 87, 897–919. 10.1146/annurev-biochem-060614-033910 29925258 PMC6535337

[B116] Wellner-Kienitz M. C. Bender K. Meyer T. Bunemann M. Pott L. (2000). Overexpressed A1 adenosine receptors reduce activation of acetylcholine-sensitive K^+^ current by native muscarinic M2 receptors in rat atrial myocytes. Circ. Res. 86, 643–648. 10.1161/01.RES.86.6.643 10746999

[B117] West R. E. Jr. Moss J. Vaughan M. Liu T. Liu T. Y. (1985). Pertussis toxin-catalyzed ADP-ribosylation of transducin. Cysteine 347 is the ADP-ribose acceptor site. J. Biol. Chem. 260, 14428–14430. 3863818

[B118] Whorton M. R. MacKinnon R. (2013). X-ray structure of the mammalian GIRK2-βγ G-protein complex. Nature 498, 190–197. 10.1038/nature12241 23739333 PMC4654628

[B119] Xue L. C. Rodrigues J. P. Kastritis P. L. Bonvin A. M. Vangone A. (2016). PRODIGY: a web server for predicting the binding affinity of protein-protein complexes. Bioinformatics 32, 3676–3678. 10.1093/bioinformatics/btw514 27503228

[B120] Yakubovich D. Rishal I. Dascal N. (2005). Kinetic modeling of Na^+^-induced, Gbg -dependent activation of G-protein-gated K^+^ channels. J. Mol. Neurosci. 25, 7–20. 10.1385/JMN:25:1:007 15781962

[B121] Yakubovich D. Berlin S. Kahanovitch U. Rubinstein M. Farhy-Tselnicker I. Styr B. (2015). A quantitative model of the GIRK1/2 channel reveals that its basal and evoked activities are controlled by unequal stoichiometry of Gα and Gβγ. PLoS Comput. Biol. 11, e1004598. 10.1371/journal.pcbi.1004598 26544551 PMC4636287

[B122] Yokogawa M. Osawa M. Takeuchi K. Mase Y. Shimada I. (2011). NMR analyses of the Gβγ binding and conformational rearrangements of the cytoplasmic pore of G protein-activated inwardly rectifying potassium channel 1 (GIRK1). J. Biol. Chem. 286, 2215–2223. 10.1074/jbc.M110.160754 21075842 PMC3023517

[B123] Zacharias D. A. Violin J. D. Newton A. C. Tsien R. Y. (2002). Partitioning of lipid-modified monomeric GFPs into membrane microdomains of live cells. Science 296, 913–916. 10.1126/science.1068539 11988576

[B124] Zhong H. Wade S. M. Woolf P. J. Linderman J. J. Traynor J. R. Neubig R. R. (2003). A spatial focusing model for G protein signals. Regulator of G protein signaling (RGS) protien-mediated kinetic scaffolding. J. Biol. Chem. 278, 7278–7284. 10.1074/jbc.M208819200 12446706

[B125] Zylbergold P. Ramakrishnan N. Hebert T. (2010). The role of G proteins in assembly and function of Kir3 inwardly rectifying potassium channels. Channels (Austin) 4, 411–421. 10.4161/chan.4.5.13327 20855978 PMC3051875

