# Supplementary figures and images for: A Collision Coupling Model Governs the Activation of Neuronal GIRK1/2 Channels by Muscarinic-2 Receptors
Source: Front Pharmacol. 2020 Aug 12;11:1216. doi: 10.3389/fphar.2020.01216 (PMC7435011; doi:10.3389/fphar.2020.01216)

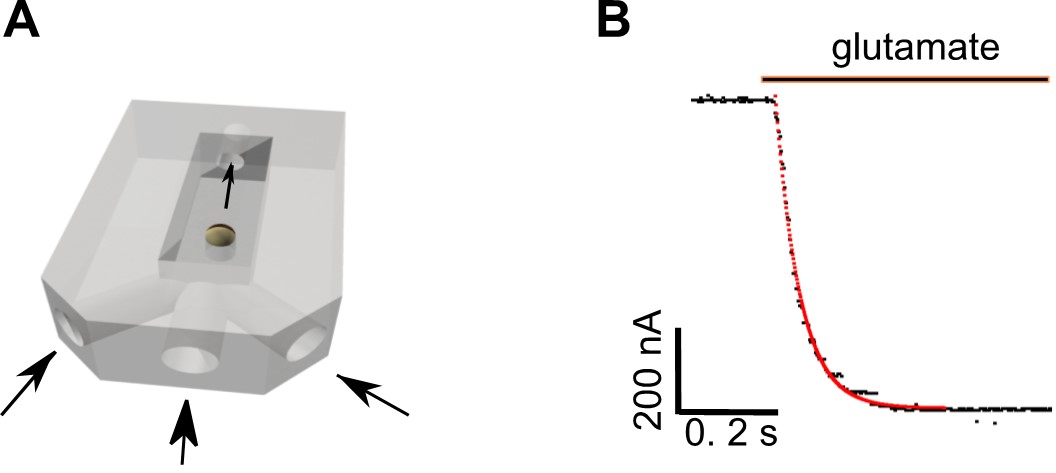

Supplement: Supplemental Figure S1 — Kinetic measurements of time course of AMPA-R activation. (A) –configuration of the experimental chamber used for fast perfusion experiments. (B)– a representative record (black dots) of current evoked by 1 mM glutamate. The red dots represent mono-exponential fit of the activation time course of the current (τact=49.5 s). [file Image_1.jpeg]

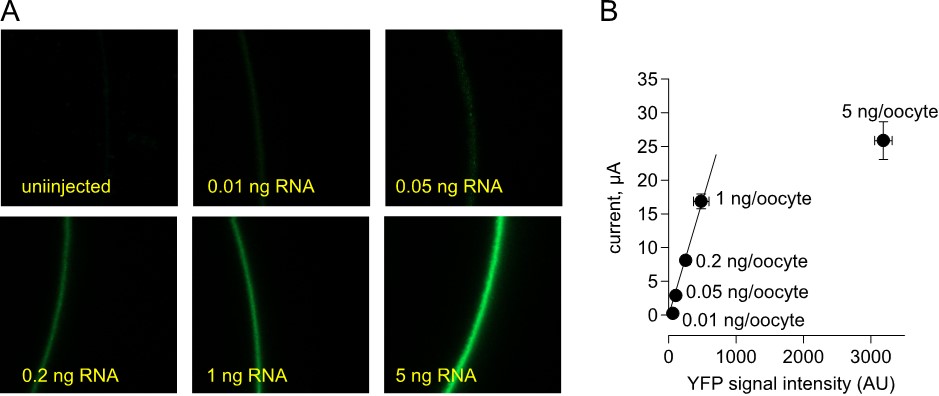

Supplement: Supplemental Figure S2 — Linear relationship between Gβγ-activated YFP-GIRK1/GIRK2 current and total surface density is observed in the range 0-1 ng of YFP-GIRK1 RNA. (A) – representative confocal images of oocytes expressing YFP-GIRK1/GIRK2. Channels were expressed by injecting the indicated doses of RNA of YFP-GIRK1 and GIRK2 (1:1) and activated by coexpression of 5 ng Gβ and 1 ng Gγ RNA. (B) – correlation between total GIRK surface density and the Gβγ-dependent GIRK current, Iβγ. Iβγ is the total agonist-independent current in Gβγ-expressing oocytes. It was measured in 24 mM K+ solution by subtracting the non-GIRK currents remaining after inhibition of >95% GIRK activity by 5 mM Ba2+ (Rubinstein et al., 2007). Total surface density is reflected in YFP fluorescence levels, functional channel density is proportional to Iβγ. Correlation between Iβγ and fluorescence is linear for RNA doses of YFP-GIRK1/GIRK2 up to 1 ng/oocyte of each subunit. n = 6-12 for each point. [file Image_2.jpeg]

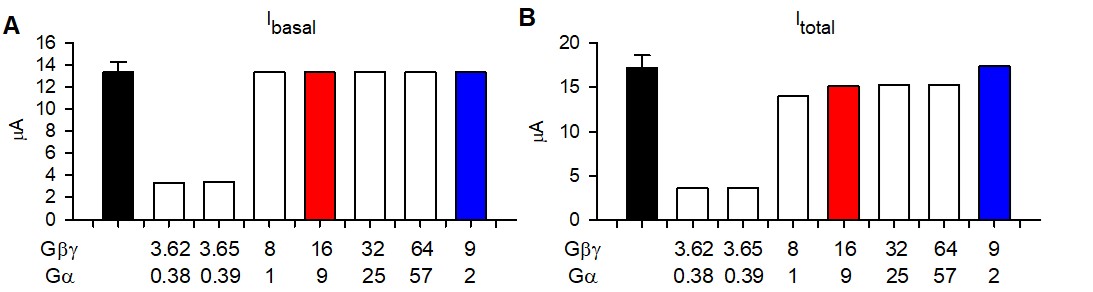

Supplement: Supplemental Figure S3 — Estimation of Gβγ and Gα densities for simulation with cooperative gating model. (A) – Ibasal currents rendered by different pairs of Gβγ and Gα densities selected for simulation. (B) – Itotal currents rendered by different pairs of Gβγ and Gα densities selected for simulation.Red bars indicate Gβγ and Gα densities used for calculations in Figures 6A, B. Blue bars indicate Gβγ and Gα densities used for calculations in Figures 6C, D. All calculations except the 9:2 Gβγ/Gα pair (blue bar) have been made for the condition of fast hydrolysis of GTP, khydrolysis=2 s-1. The 16:9 Gβγ/Gα pair was the lowest dose of G protein subunits per channel that reproduced Itotal under this condition. Lower doses of Gβγ/Gα produced lower Itotal. The 9:2 Gβγ/Gα pair was the lowest G protein subunits combination that reproduced Itotal under the condition of low GTP hydrolysis rate (khydrolysis=0.02 s-1). [file Image_3.jpeg]
