# Supplementary material for: A Collision Coupling Model Governs the Activation of Neuronal GIRK1/2 Channels by Muscarinic-2 Receptors
Source: Front Pharmacol. 2020 Aug 12;11:1216. doi: 10.3389/fphar.2020.01216 (PMC7435011; doi:10.3389/fphar.2020.01216)
Supplement: Supplementary file 4 [file DataSheet_1.docx]

## Supplemental material 1

In order to estimate the initial concentrations of G proteins which would be further utilized for kinetic simulations we assumed that GIRK-Gα-Gβγ system reaches steady-state after application of the agonist and there is complete dissociation of Gα from Gβγ . For “graded contribution” model steady-state concentrations of GIRK and Gβγ were estimated utilizing Eq. 3-10

$${Eq.3: C}_{0}+C_{1}+C_{2}+C_{3}+C_{4}=C_{\mathrm{total}}$$

$${Eq.4: C}_{1}=\frac{4\cdot C_{0}\cdot G\beta\gamma}{K_{D}}$$

$${Eq.5: C}_{2}=\frac{3\cdot C_{1}\cdot G\beta\gamma}{2\cdot K_{D}}$$

$${Eq.6: C}_{3}=\frac{2\cdot C_{2}\cdot G\beta\gamma}{3\cdot K_{D}}$$

$${Eq.7: C}_{4}=\frac{C_{3}\cdot G\beta\gamma}{4\cdot K_{D}}$$

$$Eq.8: G\beta\gamma+C_{1}+C_{2}+C_{3}+C_{4}={G\beta\gamma}_{\mathrm{total}}$$

$${Eq.9: (f}_{1}\cdot C_{1}+f_{2}\cdot C_{2}+f_{3}\cdot C_{3}+f_{4}\cdot C_{4})\cdot cf=I_{\mathrm{total}}$$

Eq.10 : cf = w$\cdot S\cdot A\cdot i$_single_$\cdot P$_o,max_

where C_o_-C_4_ are concentrations of GIRK channels bound to 0-4 Gβγ subunits, C_total_ is the total channel concentration, Gβγ_total_ is the total Gβγ concentration, f_1_-f_4_ are the contributions of C_1_-C_4_ states of channel occupancy to maximal open probability, cf is a conversion factor and I­_total_ is the current recorded on application of agonist, W is the width of interaction space (10 nm), S is the oocyte membrane area (2$\cdot$ 10^7^ µm^2^), P_o,max_ is the maximal open probability under saturating Gβγ conditions (0.105) and i_single_ is the amplitude of unitary current (~ 0.6 pA in 24 meq K+ ) and A iss the Avogadro number (Yakubovich et al., 2015). Based on estimation of Gβγ_total_, we subsequently calculated the Gα_total_ value combining Eq. 3-10 with Eq. 11-13

$$Eq.11: GaGDPG\beta\gamma+G\beta\gamma+{CG\beta\gamma}_{1}+{CG\beta\gamma}_{2}+{CG\beta\gamma}_{3}+{CG\beta\gamma}_{4}={G\beta\gamma}_{\mathrm{total}}$$

$$Eq.12: GaGDPG\beta\gamma+Ga=\mathrm{Ga}_{\mathrm{total}}$$

$${Eq.13: (f}_{1}\cdot C_{1}+f_{2}\cdot C_{2}+f_{3}\cdot C_{3}+f_{4}\cdot C_{4})\cdot cf=I_{\mathrm{basal}}$$

where I_basal_ is the current recorded in the absence of agonist. Parenthetically, assuming a finite but very low affinity of Gα_GTP_ to Gβγ, 15 µM (which we utilized for time-course simulation of agonist activation) does not significantly change the results.

A similar way to estimate initial concentrations was implemented for Touhara et al. model. Eq. 4- 9, and 13 were respectively modified to account for cooperative binding of Gβγ to GIRK rendering:

$${Eq.14: C}_{1}=\frac{4\cdot C_{0}\cdot G\beta\gamma}{K_{D}}$$

$${Eq.15: C}_{2}=\frac{3\cdot C_{1}\cdot G\beta\gamma}{2\cdot{\mu\cdot K}_{D}}$$

$${Eq.16: C}_{3}=\frac{2\cdot C_{2}\cdot G\beta\gamma}{3\cdot\mu^{2}\cdot K_{D}}$$

$${Eq.17: C}_{4}=\frac{C_{1}\cdot G\beta\gamma}{4\cdot{\mu^{3}\cdot K}_{D}}$$

$${Eq.18: C}_{4}\cdot cf=I$$

where I is respectively I_total_ or I_basal_ for calculations of Gβγ_total_ and Gα_total_.

## Supplemental material 2

For simulation of time-course of GIRK1/2 activation we generated a system of ordinary differential equation based on scheme of G-protein cycle and scheme of channel gating (Fig.4A and B).

Eq. 19: d(R)/dt =-R∙( k_1f_∙A+k_2f_∙G_gdp_) +k_1b_∙RA+k_2b_∙RG_GDP_

Eq.20: d(RG_GDP_)/dt =-RG_GDP_∙(k_3f_∙A+k_2b_)+k_3b_∙RAG_GDP_+k_2f_∙R∙G_GDP_

Eq.21: d(RAG_GDP_)/dt = -RAG_GDP_∙(k_3b_+k_5f_+k_4b_)+k_4f_∙RA∙G_GDP_+k_5b_∙RAG_0_∙GDP+k_3f_∙RG_GDP_∙A

Eq.22: d(RA)/dt = -RA∙(k_1b_+k_4f_∙G_GDP_+k_8b_∙Gα_GDP_)+k_1f_∙R∙A+k_4b_∙RAG_GDP_+k_8f_∙RAGα_GDP_

Eq.23: d(RAG_0_)/dt =-RAG_0_∙(k_5b_∙GDP+k_6f_∙GTP)+k_5f_∙RAG_GDP_ +k_6b_∙RAG_GTP_

Eq.24: d(RAG_GTP_)/dt = - RAG_GTP_∙(k_6b_+k_7f_)+k_6f_∙RAG_0_∙GTP+k_7b_∙RAGα_GTP_∙Gβγ

Eq.25: d(RAGα_GTP_)/dt = -RAGα_GTP_∙(k_8f_+k_7b_∙Gβγ)+k_7f_∙RAG_GTP_+k_8b_∙RA∙Gα_GTP_

Eq.26: d(Gα_GTP_)/dt = - Gα_GTP_ ∙(k_9f_+k_8_∙RA)+k_8f_∙RAGα_GTP_

Eq.27: d(Gα_GDP_)/dt = -k_10f_∙Gα_GDP_∙Gβγ+k_9f_∙Gα_GDP_

Eq.28: d(G_GDP_)/dt =-G_GDP_∙(k_2f_∙R+k_4f_∙RA)+k_10f_∙Gα_GDP_∙Gβγ+k_2b_∙RG_GDP_+k_4b_∙RAG_GDP_

Eq.29: d(Gβγ)/dt =-Gβγ∙(k_10f_∙Gα_GDP_+k_7b_∙RAGα_GTP_+4∙k_on_∙C_0_+3∙k_on_∙C_1_+2∙ k_on_∙C_2_+ k_on_∙C_3_)+k_7f_∙RAG_GTP_+k_10b_∙G_GDP_

+k_off_∙(C_1_+2∙C_2_+3∙C_3_+4∙C_4_)

Eq.30: d(C_0_)/dt = - 4∙k_on_∙C_0_∙Gβγ +k_off_∙C_1_

Eq.31: d(C_1_)/dt = -C_1_∙(3∙k_on_∙Gβγ+k_off_)+4∙ k_on_ ∙C_0_∙Gβγ+2∙k_off_∙C_2_

Eq.32: d(C_2_)/dt = -C_2_∙(2∙k_on_∙Gβγ+2∙k_off_)+3∙k_on_∙C_1_∙Gβγ+3∙k_off_∙C_3_

Eq.33: d(C_3_)/dt = -C_3_∙(k_on_∙Gβγ+3∙k_off_)+2∙ k_on_∙C_2_∙Gβγ+4∙k_off_∙C_4_

Eq.34: d(C_4_)/dt = -4∙ k_off_ ∙C_4_ +k_on_∙C_3_∙Gβγ

For all kinetic simulations GDP and GTP were kept constant and respectively 10 and 100 µM (Traut, 1994). A – agonist concentration – in our case ACh was assumed to step increase from 0 to 10 µM and also was kept constant.
